# The genetic map of goldfish (*Carassius auratus*) provided insights to the divergent genome evolutions in the Cyprinidae family

**DOI:** 10.1038/srep34849

**Published:** 2016-10-06

**Authors:** You-Yi Kuang, Xian-Hu Zheng, Chun-Yan Li, Xiao-Min Li, Ding-Chen Cao, Guang-Xiang Tong, Wei-Hua Lv, Wei Xu, Yi Zhou, Xiao-Feng Zhang, Zhi-Peng Sun, Shahid Mahboob, Khalid A. Al-Ghanim, Jiong-Tang Li, Xiao-Wen Sun

**Affiliations:** 1 Heilongjiang River Fisheries Research Institute, Chinese Academy of Fishery Sciences, Harbin 150070, China; 2 Centre for Applied Aquatic Genomics, Chinese Academy of Fishery Sciences, Beijing 10014, China; 3 Tianjin Fisheries Research Institute, Tianjin, 300221, China; 4 Stem Cell Program of Boston Children’s Hospital, Division of Hematology/Oncology, Boston Children’s Hospital and Dana Farber Cancer Institute, Harvard Medical School, Boston, MA 02115, USA; 5 Department of Zoology, College of Science, King Saud University, P.O. Box 2455, Riyadh 11451, Saudi Arabia

## Abstract

A high-density linkage map of goldfish (*Carassius auratus*) was constructed using RNA-sequencing. This map consists of 50 linkage groups with 8,521 SNP markers and an average resolution of 0.62 cM. Approximately 84% of markers are in protein-coding genes orthologous to zebrafish proteins. We performed comparative genome analysis between zebrafish and medaka, common carp, grass carp, and goldfish to study the genome evolution events in the Cyprinidae family. The comparison revealed large synteny blocks among Cyprinidae fish and we hypothesized that the Cyprinidae ancestor undergone many inter-chromosome rearrangements after speciation from teleost ancestor. The study also showed that goldfish genome had one more round of whole genome duplication (WGD) than zebrafish. Our results illustrated that most goldfish markers were orthologous to genes in common carp, which had four rounds of WGD. Growth-related regions and genes were identified by QTL analysis and association study. Function annotations of the associated genes suggested that they might regulate development and growth in goldfish. This first genetic map enables us to study the goldfish genome evolution and provides an important resource for selective breeding of goldfish.

The Cyprinidae family (Telestei) includes about 3,000 species and is the largest family in vertebrate. The numbers of chromosomes in this family greatly vary. For instance, bighead carp has 48 chromosomes^1^ while the chromosome number of gibel carp is up to 156^2^. Among the members in this family, genome sequences of zebrafish (*Danio rerio*)^3^, grass carp (*Ctenopharyngodon idella*)^4^ and common carp (*Cyprinus carpio*)^5^ have been published. Cross-species comparisons revealed that grass carp had a chromosome fusion in relative to zebrafish and that common carp had one more round of whole genome duplication (WGD) than zebrafish. Whether other species in the Cyprinidae family had WGDs or genome rearrangements are still unknown. Comparing more genomes in this family will help us understand the genome evolution and the genetic basis of post-speciation expansion in Cyprinidae.

Goldfish (*Carassius auratus*), one member in the Cyprinidae family, has twice the number of chromosomes (n = 50) than zebrafish (n = 25). It is speculated to have undergone one more round of WGD compared to other teleosts^6^. Teleosts are widely believed to have experienced a “fish-specific” third-round (3R) of WGD event. Therefore, this additional round of WGD was considered as the fourth-round (4R) of WGD goldfish would be a valuable model to study the consequences of genome duplication^7^. Goldfish is unique among vertebrates since it can survive for an extended period in complete absence of molecular oxygen^8^. As one of the earliest domesticated fish^9^, it has highly various morphologies and some variants are important aquarium fish. Therefore, it is also a useful model to study genetic variance as a result of domestication. These special genomic and phenotypic characteristics argue that goldfish is a suitable model to study genome duplication and physiological adaptation. Hence, establishment of goldfish genomic resources will facilitate applications of this system in many fields of studies, such as genome evolution, physiology, and neurobiology^10^. Although goldfish transcriptome was reported and would provide a resource for functional and comparative genomic analyses^11^, additional genomic resources are much needed for comprehensive genetic and genomic analysis.

In this study, we performed transcriptome sequencing of all progenies from a mapping family of goldfish. After genotyping, we constructed a high-density genetic map. The markers on the map were mainly located in protein-coding genes. We then examined gene syntenies in goldfish, three Cyprinidae fish (zebrafish, grass carp and common carp), and medaka, to study the genome evolution events in this family. Finally, we identified candidate regions and genes related to goldfish growth traits on this map using QTL analysis and association study.

## Results

### Genotype calling using RNA-Seq data

We obtained 2.1 Gb of RNA-seq reads for the male parent. After filtering out the low-quality reads, *de novo* assembly and scaffolding, we obtained 80,298 transcripts with an N50 length of 1,245 bp and total bases of 61.7 Mb (Supplementary Table S1). The length distribution of all transcripts is shown in Supplementary Fig. S1. These transcripts were used as a reference transcriptome for genotyping.

We also sequenced 172 Gb of reads for the female parent and 79 F_2_ offspring. The raw RNA-sequencing reads of all samples were deposited in the European Nucleotide Archive (ENA) under the project of PRJEB12518. We aligned cleaned RNA-seq reads to reference transcripts using BWA^12^. The overall mapping ratio to reference transcripts was about 70% (Supplementary Table S2). We then used stringent criteria to identify high-quality SNPs in all individuals. Finally, a total of 488,245 loci were genotyped, which were covered by at least 90% of individuals and heterozygous in at least one parent.

### High-density genetic map

A chi-square test identified 50,353 markers that conformed to the expected Mendelian ratios (p value > 0.01). These markers were located on 21,486 transcripts. Among them, 8,344 transcripts had a minimum of two polymorphic markers. After the number of markers was reduced by selecting the representative SNPs, JoinMap assigned 8,521 markers to 50 linkage groups at the LOD threshold of 6. The group number is consistent with the haploid chromosome number of the goldfish^13^ (Supplementary Fig. S2 and Table S3). For these mapped markers, a high depth (31.6 × depth on average) was sequenced per offspring (Supplementary Fig. S3), suggesting that these polymorphic markers were of high quality. The total map length was 5,252 cM with an average marker interval of 0.62 cM (Table 1). LG5, the largest goldfish linkage group, comprised of 185 markers in 153.5 cM; and LG39, the smallest linkage group, comprised of 119 markers in 52.3 cM. The 8,521 markers were distributed on 5,202 reference transcripts. All these marker transcripts were longer than 200 bp, 90% of which were longer than 560 bp (Supplementary Fig. S4). The long sequences were suitable for designing PCR primers, which would be applied into future selective breeding.

We performed blastx searches of goldfish reference transcripts against zebrafish proteins and found that 33,895 goldfish reference transcripts had significant sequence homology to 17,551 zebrafish genes, at a proximal 2:1 mapping ratio. Among 5,202 marker transcripts on the linkage map, 4,279 (covering 7,170 markers) had orthologous zebrafish protein-coding genes (Supplementary Table S4). The significant proportion of protein-coding markers would help us study the genome structure and evolution of goldfish.

### Discovery of different genome evolution events among Cyprinidae

**Figure 1:**
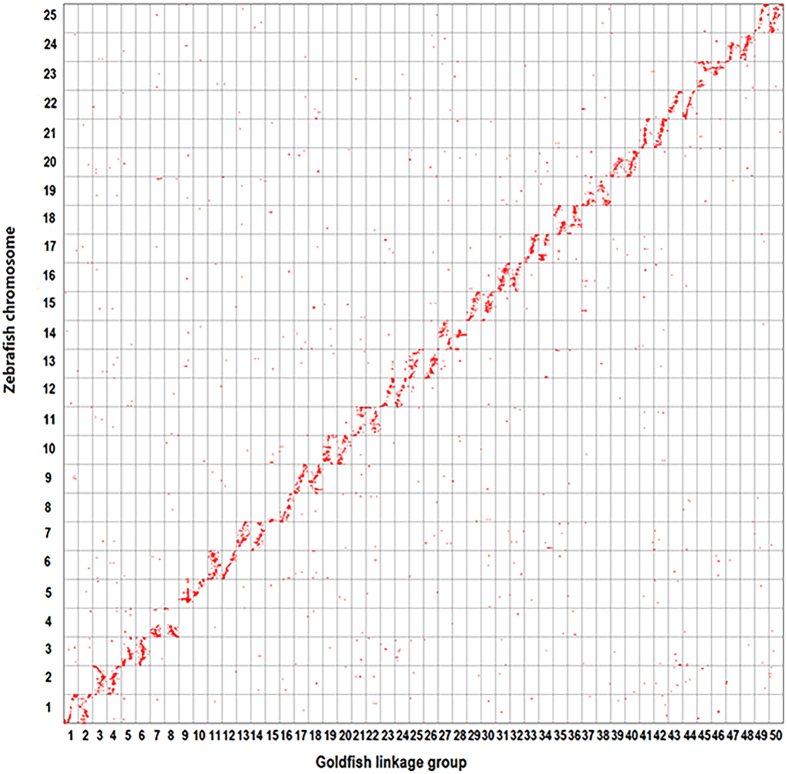
Chromosome-synteny analysis between goldfish linkage map and zebrafish genome. An orthologous gene pair between two species is represented with a red dot. Synteny comparison reveals 2:1 syntenic correspondences between the two species.

Comparative analysis among four Cyprinidae fish (zebrafish, grass carp, common carp and goldfish) and medaka was performed to investigate genome rearrangements and duplication events in the Cyprinidae family. Firstly, the orthologous gene pairs between goldfish and zebrafish were used in the following analysis (Supplementary Table S4). The synteny analysis revealed that there was a 2:1 gene-synteny mapping between 50 goldfish linkage groups and 25 zebrafish chromosomes. In general, two duplicated goldfish linkage groups were homologous to one zebrafish chromosome. The 2:1 genome-wide orthologous mapping and the 2:1 chromosome-synteny between goldfish and zebrafish supported the hypothesis that there existed one more round of whole genome duplication in goldfish than in zebrafish (Fig. 1).

**Figure 2:**
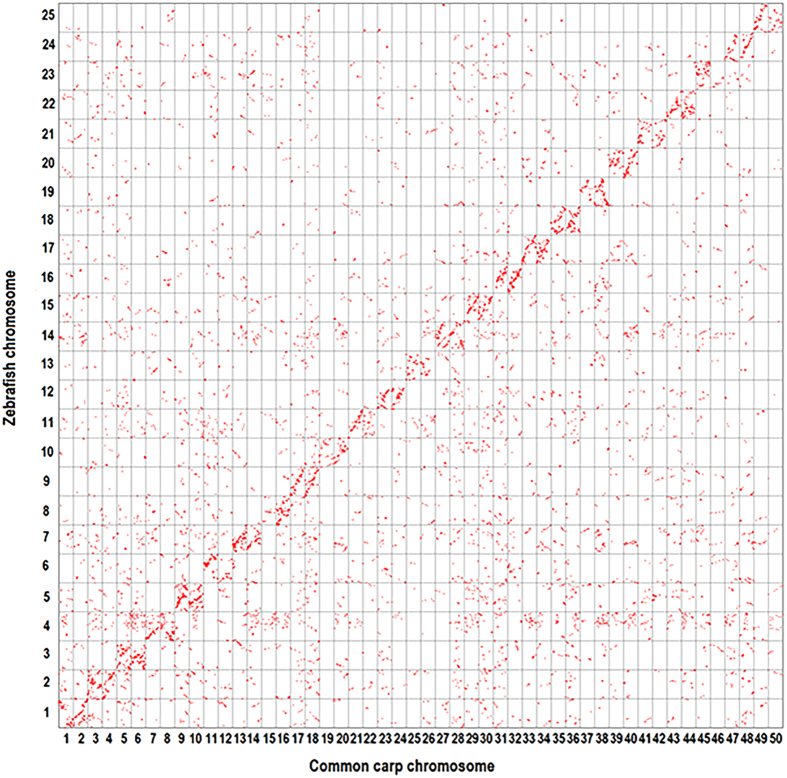
Comparative genomic analysis of common carp and zebrafish. Each red dot in the Oxford grid represents the position of an orthologous gene pair in the corresponding common carp and zebrafish genomes. In general, common carp and zebrafish chromosomes exhibit 2:1 correspondence.

Secondly, we identified 13,744 common carp proteins on 50 chromosomes had orthologous zebrafish proteins. The comparison between the common carp genome and the zebrafish genome revealed 2:1 synteny between 50 common carp chromosomes and 25 zebrafish chromosomes (Fig. 2). Although there were a number of minor chromosome rearrangements on common carp chromosomes, in general two common carp chromosomes were found homologous to one zebrafish chromosome. This observation was consistent with previous studies that there was one additional round of WGD in common carp than in zebrafish^5^,^14^,^15^ (Fig. 2).

**Figure 3:**
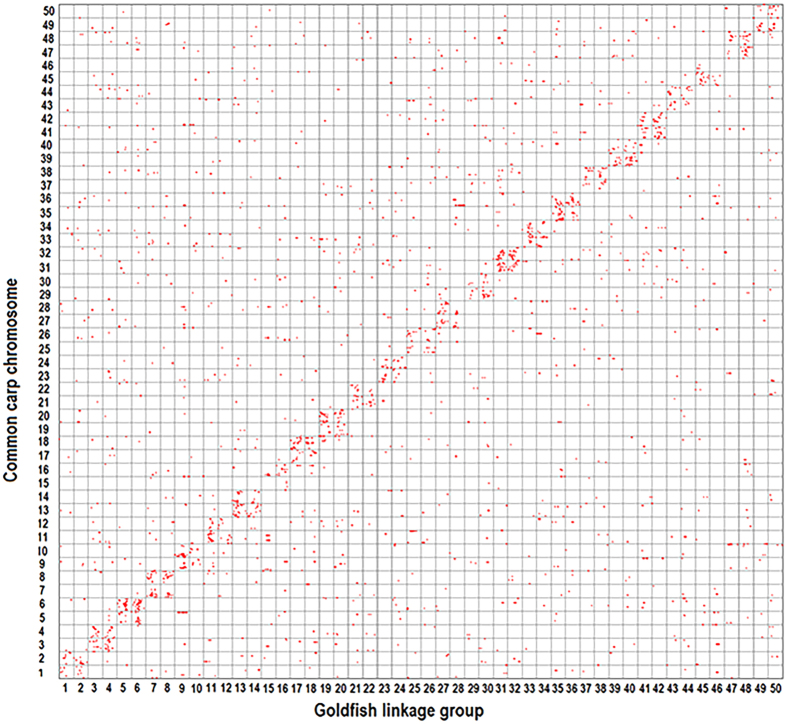
Genome-wide synteny comparisons between goldfish and common carp. Over all, two duplicated goldfish linkage groups were orthologous to two duplicated common carp chromosomes, at a 2:2 correspondence genome-wide synteny.

Thirdly, we examined gene homology and synteny between goldfish and common carp. In the above two analyses, both genomes had an additional round of WGD compared to zebrafish genome. In each species two linkage groups or chromosomes were homologous to one zebrafish chromosome with 2:1 synteny. A comparison between goldfish linkage groups and common carp chromosomes would reveal distinct genome rearrangements after their WGDs. The best-match reciprocal homolog searches identified 23,001 gene pairs between goldfish and common carp. A total of 3,520 goldfish marker transcripts (with 6,022 markers) had orthologous common carp genes. A 2:2 synteny relationship was observed between goldfish and common carp (Fig. 3), where two duplicated goldfish linkage groups were primarily syntenic to two duplicated common carp chromosomes. For instance, among 157 markers on two goldfish duplicated linkage groups (LG5 and LG6), 95 markers (61%) had orthologs on two common carp duplicated chromosomes (LG5 and LG6). The remaining markers (62) had orthologs on other common carp chromosomes. Six markers on goldfish LG5 and LG6 had orthologs on common carp LG44, indicating many minor inter-chromosome rearrangements between common carp and goldfish after their WGDs.

**Figure 4:**
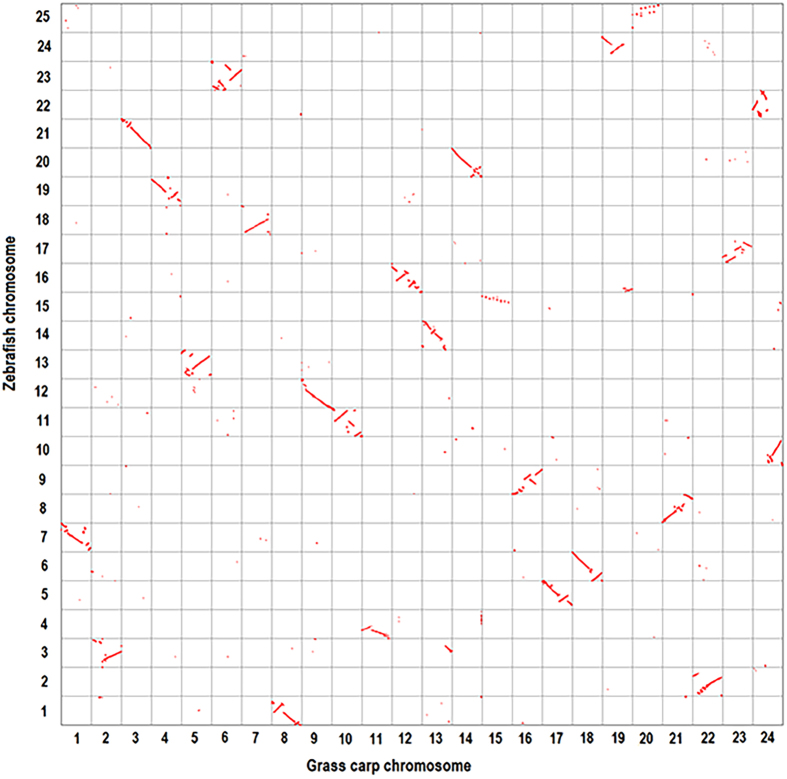
The comparison between grass carp and zebrafish genomes. The comparison reveals that three grass carp chromosomes exhibited 1:2 syntenic correspondences to six zebrafish chromosomes, indicating three putative fusion events in grass carp.

Fourthly, 13,445 one-to-one orthologous gene pairs were identified between grass carp and zebrafish on the basis of syntenic blocks by MCScanX. The Oxford grid between grass carp and zebrafish (Fig. 4) revealed that 21 of 24 grass carp chromosomes had a high degree of conserved synteny with zebrafish chromosomes. Each in the other three grass carp chromosomes (chr13, chr19 and chr24) was syntenic to two zebrafish chromosomes. There was a major genome fusion occurred in grass carp chr24, consistent with the observation of Want *et al*.^4^. This chromosome was orthologous to two zebrafish chromosomes, chr10 and chr22. We observed another two minor chromosome fusions on grass carp chr13 and chr19. Although most markers on grass carp chr13 were orthologous to genes on zebrafish chr14, a small number of genes on this chromosome had orthologs on zebrafish chr3. Likewise, grass carp chr19 had co-orthology to zebrafish chr16 and chr24. The 1:2 synteny between three grass carp chromosomes and six zebrafish chromosomes indicated that there were inter-chromosome fusion events in grass carp.

The comparisons among four Cyprinidae fish revealed that goldfish and common carp had additional round of WGD compared to zebrafish and grass carp. To further demonstrate additional round of WGD in goldfish, the genome sizes of grass carp, zebrafish, common carp and goldfish were estimated using real-time PCR^16^. The genome sizes were estimated to be 1.03 pg, 1.58 pg, 1.80 pg and 1.78 pg, respectively (Supplementary Table S5, Supplementary Fig. S5 and S6), similar as the published data^17^. The goldfish genome size is equivalent to that of common carp and approximate 1.8 times that of grass carp. Considering that grass carp is diploid and common carp is tetroploid, the result provided evidence that goldfish underwent additional WGD compared to grass carp. An ancient intron size expansion led to larger genome size of zebrafish than that of grass carp^18^. Nevertheless, since zebrafish is diploid as grass carp, zebrafish has smaller genome size than goldfish and common carp.

**Figure 5:**
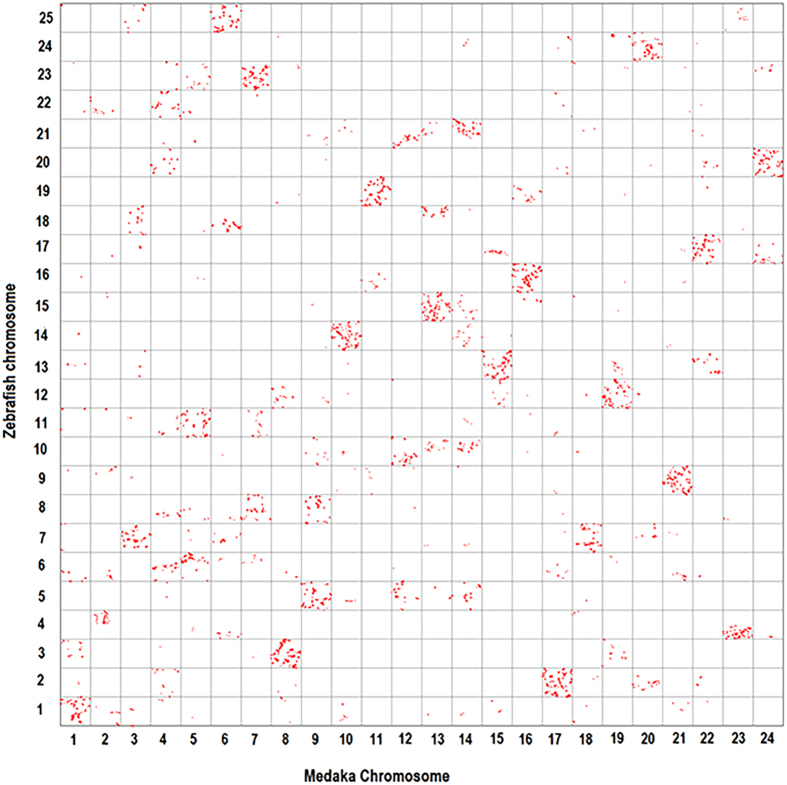
Genomic comparisons between medaka and zebrafish. Some zebrafish chromosomes exhibit 1:1, 1:2 and 1:3 correspondences to medaka chromosomes, suggesting complex genome rearrangements during speciation.

Finally, the comparison analysis between zebrafish and medaka showed that complex genome rearrangements were present in zebrafish genome after its separation from teleost ancestor (Fig. 5). We retained 9,492 one-to-one orthologous pairs for plotting. Although many zebrafish chromosomes tended to be represented on only one medaka chromosome, we observed both 1:2 and 1:3 synteny relationships between zebrafish and medaka. For instance, the majority of genes on each of chromosomes 4, 6, 7, 13, 14, 20 and 21 were homologous to genes on two medaka chromosomes. Furthermore, genes on zebrafish chromosomes of 5, 8, 10, 17 and 18 had orthologs that were distributed broadly among three medaka chromosomes.

Taken together, our genome-wide comparative analysis revealed a high degree of gene synteny among Cyprinidae fish after the speciation of this family from teleost ancestor. We also observed different genome evolution events in the Cyprinidae family. These events might explain diverse phenotypes of members in this largest vertebrate family.

### QTL analysis of growth traits

**Figure 6:**
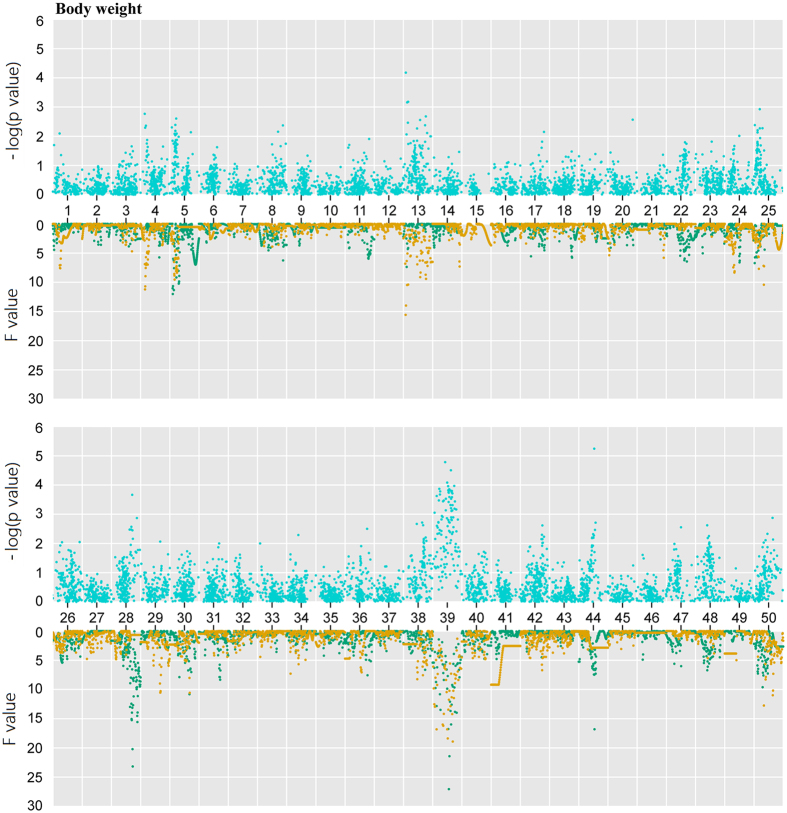
BW-related regions in goldfish. Association analysis (blue points) and QTL mapping were carried out for BW. In QTL scanning, green dots represent sire-based QTL F values and yellow dots are dam-based QTL F values.

**Figure 7:**
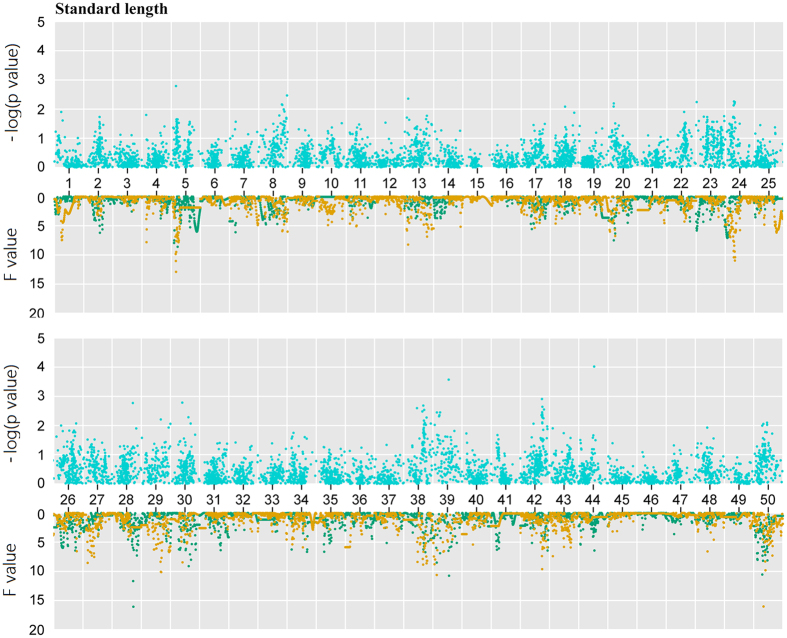
SL-related regions in goldfish. Association analysis (blue points) and QTL mapping were carried out for SL. In QTL scanning, green dots and yellow dots represent sire-based QTL F values and dam-based QTL F values, respectively.

Pearson’s correlation analysis between BW and SL revealed that they were statistically significantly correlated (correlation coefficient of 0.83, p value of 2.2 × e^−16^), indicating that these traits might be regulated by a common set of genes. We performed QTL mapping to identify trait-related markers and genes. Firstly, the F values of dam-based analysis and sire-based mapping showed similar distributions (Table 2, Fig. 6). For BW, the dam-based analysis identified nine significant QTLs. One QTL at 36 cM on LG 39 had the highest F value of 18.91 with a 95% of CI from 3 cM to 40 cM. The sire-based mapping identified six significant loci, where the 95% CIs of four loci overlapped the ones of four dam-based loci. For SL, the dam-based analysis identified five significant loci (Table 2 and Fig. 7). One QTL at 40 cM on LG50 had the highest F value of 16.03 with a 95% CI between 37 cM and 82 cM. The sire-based QTL mapping identified three significant QTL regions, two of which had the overlapping 95% CIs with the dam-based CIs.

Secondly, comparing the QTL regions for BW and SL revealed high proportion of overlapping QTLs between BW and SL (six out of eight QTL regions for SL, Table 2). In the dam-based QTL genome scanning, the 95% CIs of three QTLs for SL were significantly related to BW. In the sire-based QTL mapping, all QTLs of SL were consistent with the ones of BW. Furthermore, we identified 180 transcripts (with 237 markers) in the flanking regions of BW-related QTL regions (Supplementary Fig. S7). A total of 135 transcripts (with 167 markers) were located in the flanking regions of SL-related QTL regions (Supplementary Fig. S7). Among the 135 SL-related transcripts, 101 transcripts (with 127 markers) were covered in the franking regions of BW-related QTLs (Supplementary Table S6 and Fig. S7). The high proportion of overlapping genes and markers was consistent with the correlation analysis, suggesting that BW and SL might be regulated by a common set of genes.

The association tests using PLINK also showed similar distribution of p values for BW and SL (Figs 6 and 7, and Supplementary Table S7). We identified 131 SNPs significantly associated with BW and/or SL. Most of SNPs (71%, 93 out of 131) were covered in the 95% CIs of QTL regions for two traits, generally supporting the QTL mapping results.

Further, we studied the functions of genes in QTL regions. Among the transcripts in the flanking regions of BW-related and SL-related QTL regions, Blast2GO assigned GO annotations to 123 BW-related transcripts and 93 SL-related transcripts (Supplementary Table S8). The GO annotations of these genes included the processes of developmental process, anatomical structure formation and growth (Supplementary Fig. S7), indicating that these genes might be involved in growth regulation. The function of orthologs in other species would help us understand the mechanisms of growth-related traits in goldfish. For instance, connective tissue growth factor (*CTGF*, marker accession of 000014952_1991) was identified to be significantly associated with goldfish BW and SL by QTL mapping. Previous studies showed that *CTGF* down-regulated the bone development^19^,^20^,^21^. In *CTGF*-null mice, multiple skeletal dysmorphisms were resulted from impaired growth plate chondrogenesis, angiogenesis, and bone formation, suggesting the physiological significance of *CTGF* in development^22^. The GO annotations of these transcripts and the participation of orthologs in growth and development suggested the significant association between these genes and goldfish growth traits. The identified growth-related genes and markers may be applied into future selective breeding of goldfish.

## Discussion

A genetic map is widely applied into the genetics and genomics studies^23^. In this study, we generated the first high-density linkage map of goldfish using SNPs. This genetic map consists of 8,521 markers, with an average resolution of 0.62 cM. This map provided sufficient resolution for QTL mapping. The other feature of this map is high proportion (84%) of protein-coding genes among all markers, which enables us to perform a genome-wide comparative analysis and discover the trait-related genes.

These features of this genetic map allowed us to perform chromosome-level comparative analysis in the Cyprinidae family and provided evidence for an additional round of WGD in goldfish. Firstly, the large-scale synteny in members of the Cyprinidae family and the presence of 1:1, 1:2 and 1:3 synteny correspondences between zebrafish and medaka indicated that complex genome rearrangements took place in Cyprinidae ancestor after its separation from other teleosts. Secondly, the comparison revealed different types of genome evolutions including fusions, fission, and duplication occurred in the family, which might be responsible for speciation expansion in Cyprinidae and substantial phenotype diversity among members. Thirdly, goldfish chromosomes showed 2:1 synteny to zebrafish chromosomes, providing evidence for the fourth round of WGD in goldfish. Fourthly, comparison between common carp genome and goldfish revealed a 2:2 correspondence. Li *et al*. proposed that the fourth round of WGD occurred in a common ancestor of common carp and goldfish^6^. On the basis of our results, we proposed that after separated from the ancestor common carp and goldfish experienced different genome rearrangement processes but in general both kept the duplicate genome components.

Furthermore, we used this high-resolution genetic map to detect growth-related markers and genes. QTL mapping and association study were performed together to identify genetic loci responsible for these traits. GO annotations provides hints that the identified genes might regulate growth. The markers form a valuable resource for the future marker-assisted selection. This map can be potentially used to analyze other important traits, including traits of hypoxia tolerance and other environmental adaptabilities.

In conclusion, we identified SNPs in a mapping family using RNA-seq and constructed a high-resolution genetic map of goldfish. To our knowledge, this is the most comprehensive genetic map to date for this important species. Using this map, we revealed divergent genome evolutions in the Cyprinidae family. Through QTL mapping analysis based on this map, we identified QTL regions and markers significantly related to BW and SL. The markers can potentially aid growth breeding of goldfish. The high-density linkage map will facilitate the genome-wide comparative genomics analysis and the mapping of phenotypes in goldfish.

## Methods

### Ethics Statement

All experiments involving the handling and treatment of fish in this study were approved by the Animal Care and Use committee of Heilongjiang River Fisheries Research Institute of Chinese Academy of Fishery Sciences (HRFRI). The methods were carried out in accordance with approved guidelines.

### Mapping family and sequencing

Two parents and 79 F_2_ individuals of one full-sib goldfish family were selected for study. This family was constructed at Hulan Experiment Station of HRFRI, Harbin, Helongjiang Province, China. Growth-related traits including body weight (BW) and standard length (SL) were measured for all progenies.

For each individual, twelve organs including scale, skin, muscle, eye, brain, liver, kidney, spleen, heart, blood, intestine and gonad were collected. Total RNAs were extracted from each tissue separately using Trizol reagent (Invitrogen, CA, USA), and then treated with RNase-free DNaseI (NEB, MA, USA) to remove genomic DNA. Equal quantities of total RNAs from each tissue were mixed. The integrity of pooled RNAs was analyzed on a Bioanalyzer 2100 machine (Agilent, CA, USA). About 3 μg of pooled RNA from each individual was used for RNA-seq library construction.

For each sample, a sequencing library with an insert size about 300 bp was constructed with TruSeq RNA Sample Preparation Kit (Illumina, CA, USA). The library was sequenced on the HiSeq2000 platform with the 2 × 100 bp mode at Berry Genomics Co., Ltd (Beijing, China).

### Constructing reference gene set and calling SNPs

For each individual, the raw transcriptome reads were processed using SolexaQA^24^ to filter low-quality reads. The high-quality reads of male parent were assembled using Trinity^25^ with default parameters. We selected the longest transcript to represent each gene and then subjected the selected transcripts to SSPACE^26^ for scaffolding. The scaffolding would elongate the transcripts. The scaffolded transcripts were used as the reference set for further genotyping.

The cleaned RNA-seq reads of two parents and 79 F_2_ progenies were aligned to the reference transcripts using BWA^12^ with default parameters. Considering that the high sequence similarity of duplicated genes might lead to multiple alignment of sequencing reads, we identified SNPs based on a subset of uniquely aligned reads. SAMtools^27^ was used to call variants. We retained high-quality of SNPs which had a minimal sequencing depth of five and a minimal mapping quality of 20. Those SNPs homozygous in two parents or absence in more than 10% of the offspring were removed.

### Constructing linkage map and annotating the coding marker transcripts

The retained markers were considered to be of high quality and included for further analysis. We performed a chi-square test to identify markers conforming to the expected Mendelian ratio (p value > 0.01). The markers that satisfied the Mendelian segregation were subjected to JoinMap^28^ to construct a linkage map. Because JoinMap could only process less than 5,500 markers at a time^29^, we adopted three strategies to reduce the computational time. Firstly, if multiple markers in a transcript had identical genotypes, we selected the marker genotyped in the most individuals to represent these markers. Secondly, for a short transcript (<500 bp in length) that harbored multiple markers, only one marker sequenced in the most individuals was selected to represent this locus. Thirdly, we assigned markers into different linkage groups (LGs) using a single-linkage clustering algorithm with a pair-wise modified independent logarithm of odds (LOD) score^30^. The LOD threshold of grouping was set as 6. In each LG, the recombination rate and map distances between markers were calculated using JoinMap with the parameters of CP population type (cross pollinator, or full-sib family), the Kosambi mapping function^31^ and the regression mapping algorithm.

The markers were developed by RNA-seq sequencing, suggesting that a significant proportion of them were from protein-coding genes. Identifying the protein-coding markers would help us perform the comparative analysis and detect the trait-associated genes. All goldfish reference transcripts were aligned against zebrafish proteins from Ensembl database^32^ using blastx^33^ with an e-value cutoff of 1 × e^−5^. For each marker transcript, we selected the best-aligned zebrafish gene.

### Comparative genomic analysis among Cyprinidae fish and medaka

To study the genome evolution events among Cyprinidae fish, we performed comparative analysis among four Cyprinidae fish (zebrafish, grass carp, common carp and goldfish) and medaka. Zebrafish genome was selected as reference since this genome has been well annotated and is almost finished^3^. Firstly, goldfish genetic map was compared with zebrafish genome. To facilitate further comparative analysis with zebrafish genome, we ordered goldfish linkage groups based on the marker similarity to zebrafish proteins. Due to one more round of WGD, goldfish has twice the number of chromosomes (n = 50) than zebrafish (n = 25). If a majority of markers on one goldfish LG were orthologous to a zebrafish chromosome, this LG was named as either (2***n***−1) or (2***n***), where ***n*** was the zebrafish chromosome number. Based on the association of goldfish transcripts to zebrafish orthologs, we constructed an oxford grid^34^ by placing all goldfish coding markers according to their orders in the genetic map on the horizontal axis and plotting zebrafish orthologs on the vertical axis.

Secondly, we compared common carp genome against zebrafish genome. The updated common carp genes were downloaded from CarpBase (www.carpbase.org). All-against-all protein alignments were performed using blastp, with an e-value cutoff of 1 × e^−5^. The alignments were subjected to MCScanX^35^ to identify syntenic blocks between two species. Two chromosome regions with the gap size set to 15 genes and at least five genes were considered to be syntenic^5^. For each common carp gene, we selected the best-aligned zebrafish gene. Common carp genes and zebrafish genes were plotted on the horizontal axis and the vertical axis of a grid, respectively.

Thirdly, we investigated the homologous and syntenic relationship between common carp and goldfish. The reciprocal blastx searches were performed using goldfish reference transcripts as queries against common carp proteins with an e-value cutoff of 1 × e^−5^. Two sequences were defined as orthologs if each of them was the best hit of the other. Then the pairs between goldfish marker transcripts and the corresponding common carp orthologs was plotted on a grid based on the position of each gene.

Fourthly, we compared grass carp genome against zebrafish genome. Grass carp proteins and the corresponding locations were downloaded from the National Center for Gene Research website (http://www.ncgr.ac.cn/grasscarp/). The blastp alignment and MCScanX searches were performed following the above criteria to build syntenic blocks. Both grass carp and zebrafish had species-specific gene duplications^3^,^4^, which might result in one-to-many orthologous pairs. We retained the reciprocal best-match ortholog pairs for each grass carp gene and zebrafish gene from the syntenic blocks, to ensure that each gene of a species had only one best-aligned hit in the other species. On an oxford grid, grass carp genes were plotted on the horizontal axis based on their orders in genome and zebrafish orthologs were on the vertical axis.

Finally, we examined the syntenic relation between zebrafish and medaka. Since the medaka genome is considered to preserve the teleost ancestor karyotype^36^, comparison between medaka and zebrafish genomes will reveal the genome rearrangement in zebrafish after its speciation from teleost ancestor. Medaka proteins were obtained from Ensembl database. After syntenic blocks were identified using MCScanX, only one-to-one orthologous pairs were retained in the following analysis. Medaka genes were plotted on the horizontal axis of an oxford grid and the zebrafish orthologs were on the vertical axis.

### Estimation of genome sizes

We adopted the strategy of Wilhelm *et al*.^16^ to estimate the genome sizes of grass carp, zebrafish, common carp, and goldfish. In brief, we designed two sets of gene-specific primers including the outer primers and the inner primers (Supplementary Table S9, Supplementary Fig. S8). The outer primers were used in the first round of PCR to prepare the standard templates in the second round of real-time PCR. During the following round of real-time PCR with the inner primers, standard curves were drawn using diluted standard templates of different concentrations. Then the genomic DNA of target species was used as template in the real-time PCR with the above volume and protocol. We compared the amplification curve with the standard curves to estimate and then calculated the genome size based on the quantified concentration and copies. The performance details were described in Supplementary Method.

### QTL mapping and association analyses of growth traits

QTL mapping analysis was performed for two growth traits, BW and SL, using GridQTL (http://www.gridqtl.org.uk/) with the regression-interval mapping method^37^. Considering different recombination frequencies between sire and dam, we identified QTL regions using sire-based and dam-based half-sib analysis, respectively. For each analysis, F value was calculated at 1 centiMorgans (cM) interval on each LG. To identify the significant QTL regions, the chromosome-wide threshold was determined using an empirical permutation method^38^ with 10,000 permutations. If the F value of one region was larger than the chromosome-wide threshold at p value < 0.05, we considered it to be a significant QTL region. Secondly, the 95% confidence interval (CI) of a QTL region was calculated using a bootstrapping algorithm^39^ with 1,000 sampling. The marker transcripts in two 5-cM flanking regions of QTL peaks were selected to study the functions of genes related to traits.

The association analyses were performed between genotypes and two traits using PLINK^40^, as a complementary approach to QTL mapping. To determine the threshold to identify the significantly associated SNPs, we firstly detected the independent SNPs^41^. We calculated linkage disequilibrium for the F_2_ population according to *r*^*2*^ value with the window width of 50 SNPs and the stepwise distance of five SNPs. An *r*^2^ threshold of 0.5 was set to detect independent SNPs^42^. We identified 171 independent SNPs. Secondly, the suggestive significance threshold was set as 5.8 × 10^−3^ (1/171). Markers with p values ≤ 5.8 × 10^−3^ were considered to be significantly associated with growth traits.

To annotate the functions of the growth-associated genes, we searched their orthologs by blastx against NCBI NR database with an e-value threshold of 10^−5^. Then we used Blast2GO^43^ with default parameters to assign the Gene Ontology (GO) annotations to gold transcripts. The GO distributions were plotted using WEGO^44^.

## Additional Information

**How to cite this article**: Kuang, Y.-Y. *et al*. The genetic map of goldfish (*Carassius auratus*) provided insights to the divergent genome evolutions in the Cyprinidae family. *Sci. Rep.* **6**, 34849; doi: 10.1038/srep34849 (2016).

## Supplementary Material

Supplementary Information

Supplementary Figure S2

Supplementary Table S2

Supplementary Table S3

Supplementary Table S4

Supplementary Table S6

Supplementary Table S7

Supplementary Table S8

## Figures and Tables

**Table 1:** Characteristics of the goldfish linkage map.

LG	Marker number	Annotated markers	Length (cM)	Average interval
LG1	156	132	118.24	0.76
LG2	164	142	124.19	0.76
LG3	187	140	100.51	0.54
LG4	200	158	127.70	0.64
LG5	185	152	153.37	0.83
LG6	171	170	113.86	0.67
LG7	159	125	90.50	0.57
LG8	149	123	99.44	0.67
LG9	256	231	85.33	0.33
LG10	128	98	96.87	0.76
LG11	207	184	91.21	0.44
LG12	136	123	121.06	0.89
LG13	189	143	80.03	0.42
LG14	143	113	142.82	1.00
LG15	86	72	79.84	0.93
LG16	148	127	91.32	0.62
LG17	174	137	119.40	0.69
LG18	251	231	113.17	0.45
LG19	219	173	119.17	0.54
LG20	162	130	83.23	0.51
LG21	146	130	79.09	0.54
LG22	124	116	103.16	0.83
LG23	214	191	119.44	0.56
LG24	146	123	133.26	0.91
LG25	197	184	108.83	0.55
LG26	192	152	120.53	0.63
LG27	163	139	97.38	0.60
LG28	219	164	150.99	0.69
LG29	145	117	91.08	0.63
LG30	185	141	110.26	0.60
LG31	233	195	122.84	0.53
LG32	141	129	82.78	0.59
LG33	161	142	121.17	0.75
LG34	192	182	122.63	0.64
LG35	214	184	100.76	0.47
LG36	172	142	94.00	0.55
LG37	125	105	78.36	0.63
LG38	142	106	91.00	0.64
LG39	119	100	52.30	0.44
LG40	153	132	94.02	0.61
LG41	247	215	103.16	0.42
LG42	253	202	122.09	0.48
LG43	161	127	119.64	0.74
LG44	143	118	144.89	1.01
LG45	121	101	78.08	0.65
LG46	191	148	72.21	0.38
LG47	131	119	72.22	0.55
LG48	154	134	110.51	0.72
LG49	98	82	88.25	0.90
LG50	169	146	116.22	0.69
Total	8,521	7,170	5,252.37	0.62

**Table 2:** QTL mapping of BW and SL in goldfish.

Trait	LG	Peak position (cM)	Sire F value	Dam F value	CI (95%)
BW	4	20		11.22	20–105
	5	17	12.01		16–148
	5	27		9.54	9–48
	13	6		15.58	6- 75
	25	38		10.42	8–103
	28	113	23.18		99–150
	29	62		10.54	24–92
	30	76	10.82		20–85
	30	77		10.57	28–109
	39	29	27.06		6–40
	39	36		18.91	3–40
	41	7		9.19	7–37
	44	81	16.81		1–105
	50	35	9.61		11–89
	50	40		12.75	31–110
SL	5	27		12.90	15–48
	24	45		10.97	13–118
	28	113	16.07		56–132.5
	29	63		10.14	36.5–92
	39	7		10.63	3–44
	39	29	10.76		1–43
	50	35	10.56		10–66
	50	40		16.03	37–82
