# Supplementary material for: The genetic map of goldfish (Carassius auratus) provided insights to the divergent genome evolutions in the Cyprinidae family
Source: Sci Rep. 2016 Oct 6;6:34849. doi: 10.1038/srep34849 (PMC5052598; doi:10.1038/srep34849)
Supplement: Supplementary Information [file srep34849-s1.doc]

**The genetic map of goldfish (*Carassius auratus*) provided insights to the divergent genome evolutions in the Cyprinidae family**

You-Yi Kuang^1,#^, Xian-Hu Zheng^1,#^, Chun-Yan Li^2,3^, Xiao-Min Li^2^, Ding-Chen Cao^1^, Guang-Xiang Tong^1^,Wei-Hua Lv^1^, Wei Xu^1^, Yi Zhou^4^, Xiao-Feng Zhang^1^, Zhi-Peng Sun^1^, Shahid Mahboob^5^, Khalid A. Al-Ghanim^5^, Jiong-Tang Li^2,*^, and Xiao-Wen Sun^1,*^

**Affiliations**

^1^ Heilongjiang River Fisheries Research Institute, Chinese Academy of Fishery Sciences, Harbin 150070, China, ^2^ Centre for Applied Aquatic Genomics, Chinese Academy of Fishery Sciences, Beijing 10014, China, ^3^ Tianjin Fisheries Research Institute, Tianjin, 300221, China, ^4^Stem Cell Program of Boston Children’s Hospital, Division of Hematology/Oncology, Boston Children's Hospital and Dana Farber Cancer Institute, Harvard Medical School, Boston, MA 02115, USA, ^5^ Department of Zoology, College of Science, King Saud University, P.O. Box 2455, Riyadh 11451, Saudi Arabia;

^#^ These authors contributed equally to this work.

*To whom correspondence should be addressed. E-Mail: lijt@cafs.ac.cn; Tel.: +86-10-6867-3905; Fax: +86-10-6869-7522. E-Mail: sunxw2002@163.com; Tel: +86-451-84869341, Fax: +86-451-84604803

**Supplementary Method**

**DNA extraction**

Genomic DNA was extracted from blood (goldfish and common carp) or caudal fin (grass carp and zebrafish) using QIAGEN DNeasy Blood & Tissue Kit (QIAGEN, Germany), and was quantified using Nanodrop 8000(Thermo Scientific, USA).

**Primers**

For zebrafish and grass carp, *tp53* gene was chosen for designing primers, *tp53* gene is a single copy gene in zebrafish, and *tp53* was found that only one copy existed in grass carp by comparing zebrafish *tp53* mRNA sequence (NM_001271820) to grass carp genome[^1^](#_ENREF_1). Initially, we chose *tp53* gene to design primers for common carp and goldfish, but failed to obtain the specific PCR products. To search single copy genes in common carp and goldfish, we carried out gene family analysis and comparative genomic analysis among zebrafish [^2^](#_ENREF_2), grass carp[^1^](#_ENREF_1), common carp [^3^](#_ENREF_3) and goldfish(data unpublished), according to these analysis results, we finally selected single copy gene *opn1sw1* for common carp, *ogg1* for goldfish. The partial sequences for these genes were listed in Supplementary Fig. S8. Primers were designed using Primer3 program [^4^](#_ENREF_4), listed in Supplementary Table S9.

**Preparation Standard DNA**

We estimated the genome size of target species following the strategy of Wilhelm *et al* [^5^](#_ENREF_5)*.* The method involves two rounds of amplification with two sets of gene-specific primers (Supplementary Table S9). The first round of PCR, named as the conventional PCR, amplifies the genomic DNA to prepare standard DNA with the outer primers. The outer primers contained the binding sites for the inner primers, which were used in the second round of real-time PCR. The conventional PCR was carried out in a total volume of 25 μL, containing 50 ng ~100 ng genomic DNA, 1 × reaction buffer(10 mM Tris-Cl (pH 8.3), 50 mM KCl, 1.5 mM MgCl2), 200 μM dNTP, 0.5 μM each gene-specific forward and reverse outer primers(Supplementary Table S9), and 2.5 U Taq DNA polymerase (Takara, Japan). The conventional PCR was performed under the following condition: 95°C for 5 min, 35 cycles with 30 s at 95°C, 30 s at 62°C and 1 min at 72°C. The products were purified using the QIAquick PCR purification kit (QIAGEN, Germany). The products were examined by gel electrophoresis to check specific amplification (Supplementary Figure S5).

**Real-time PCR**

The purified products were quantified using Nanodrop 8000 (Thermo Scientific, USA) and then diluted to a serial concentrations from 10^2^ to 10^10^ copies. To draw standard curves, the diluted products of different concentrations were used as the templates for the second round of real-time PCR. All read-time PCR reactions were run in triplicate. The real-time PCR was performed in a volume of 20 μL on an ABI7500 QPCR System. The PCR mixture consisted of 2 µL templates, 0.4 µM each forward and reverse gene-specific inner primers(Supplementary Table S9), 10 µL of SYBR Premix Ex Taq II (Takara, Japan, including *Taq* DNA polymerase, reaction buffer, and deoxynucleotide triphosphate mixture), and 0.4 µL ROX Reference Dye II. The cycling protocol was as follows: initial denaturation for 30 s at 95 °C, 40 cycles with 5 s at 95 °C, 34 s at 63.5°C or 65 °C (Supplementary Table S9). Melt curve stage consisted of 15 s at 95 °C, 1 min 60 °C and 15 s at 95 °C. Then the standard curves were drawn based on the amplifications with standard templates of different concentrations.

The genomic DNA of target species was quantified using Nanodrop 8000 (Thermo Scientific, USA) and used as template in the following real-time PCR with the above volume and protocol. We estimated the corresponding copies by comparing the amplification curve with the standard curves and then calculated the genome size based on the quantified concentration and copies according to Wilhelm and Hahn [^6^](#_ENREF_6).

**Supplementary Figure S1. The length distribution of reference transcripts**


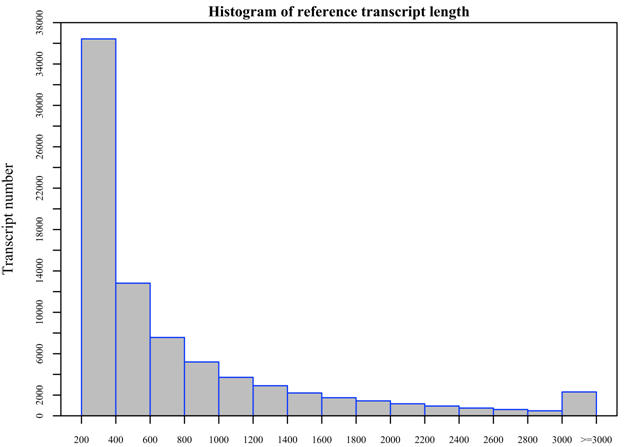


**Supplementary Figure S2. Linkage groups of goldfish.**

Markers are listed on both sides of the linkage groups.

(Included in a separated pdf file)

**Supplementary Figure S3. Sequencing depths of markers in 81 samples.**

We aligned the clean RNA-seq reads of each sample to reference transcripts using BWA[^7^](#_ENREF_7) and then calculated the sequencing depths of all mapped SNPs in each sample. X axis shows the sequencing samples and Y axis shows the distribution of sequencing depth of all markers in each sample. The error bars represent the standard variance of sequencing depths. S and D are sire and dam, respectively. The others are offsprings of the mapped family.





**Supplementary Figure S4. Length distribution of marker reference transcripts.**


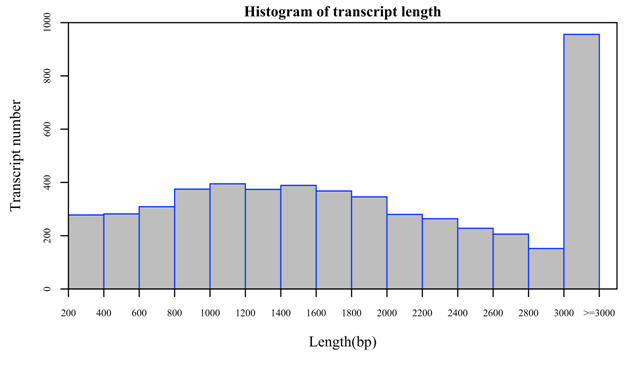


**Supplementary Figure S5. Gel electrophoresis of conventional PCR and real-time PCR products for genome size estimation.** The PCR products were detected using 2% agarose gel. The results revealed gene-specific amplifications. C: conventional PCR product; R: real-time PCR product, M: DNA molecular weight marker.

**
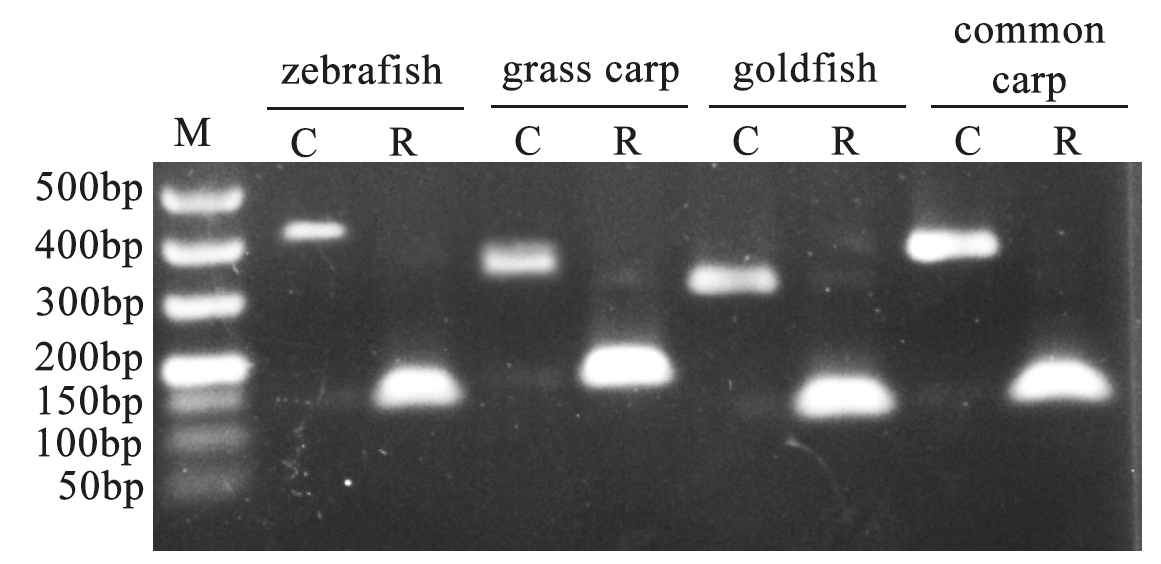
**

**Supplementary Figure S6. Signal curves in real-time PCR.** The curve marked with ‘DNA’ represents the signal using genomic DNA of target species as template. The other curves are signals using diluted products amplified by the conventional PCR as standard templates. ∆Rn means normalized signal.

**
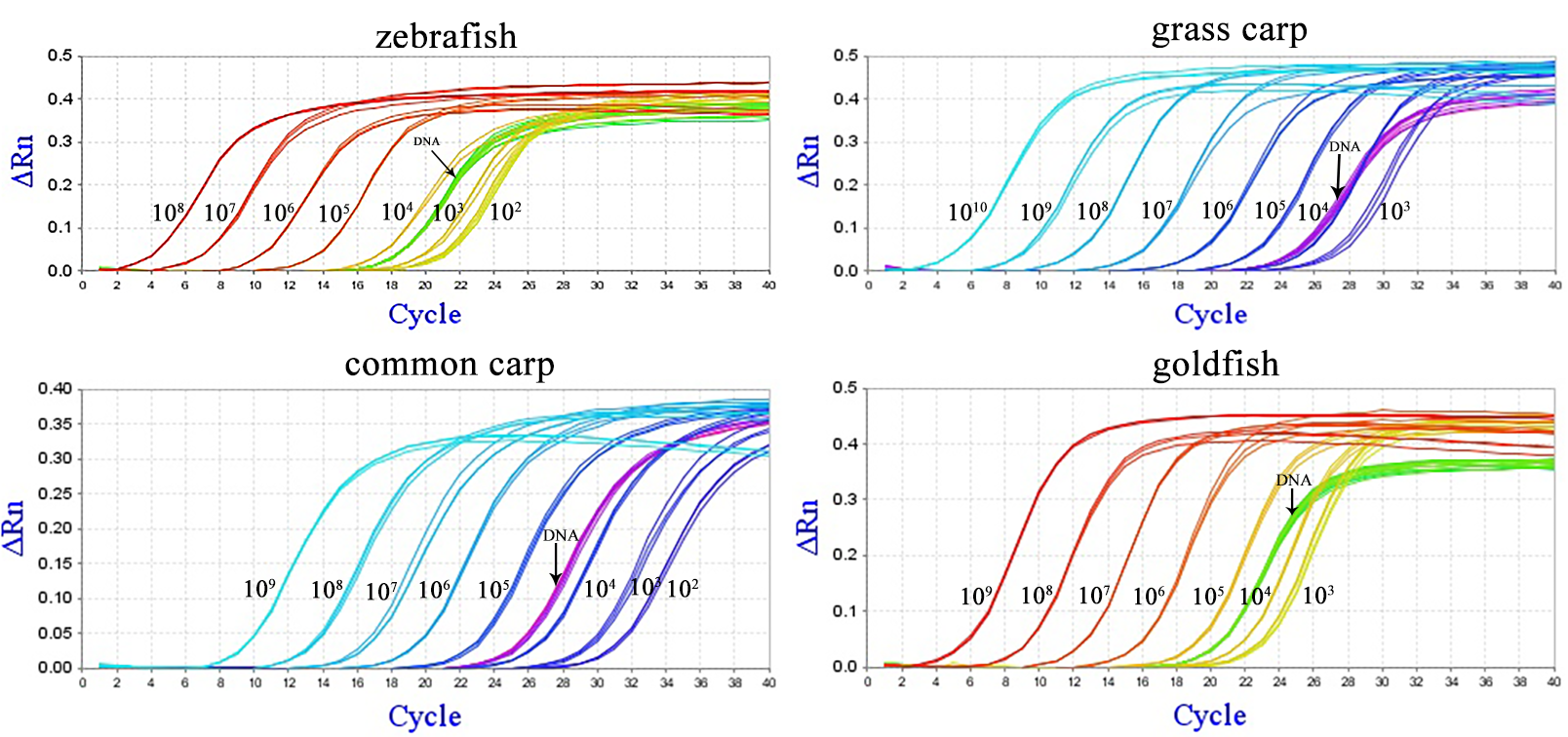
**

**Supplementary Figure S7. The GO function distribution of genes associated with BW and SL.**

The significantly associated markers (A) and genes (B) were illuminated by venn diagrams for BW and SL. Markers and genes within two 5-cM flanking regions of QTL peaks were selected (Supplementary Table S5). The GO term annotations of genes (Supplementary Table S7) were plotted using WEGO (C).


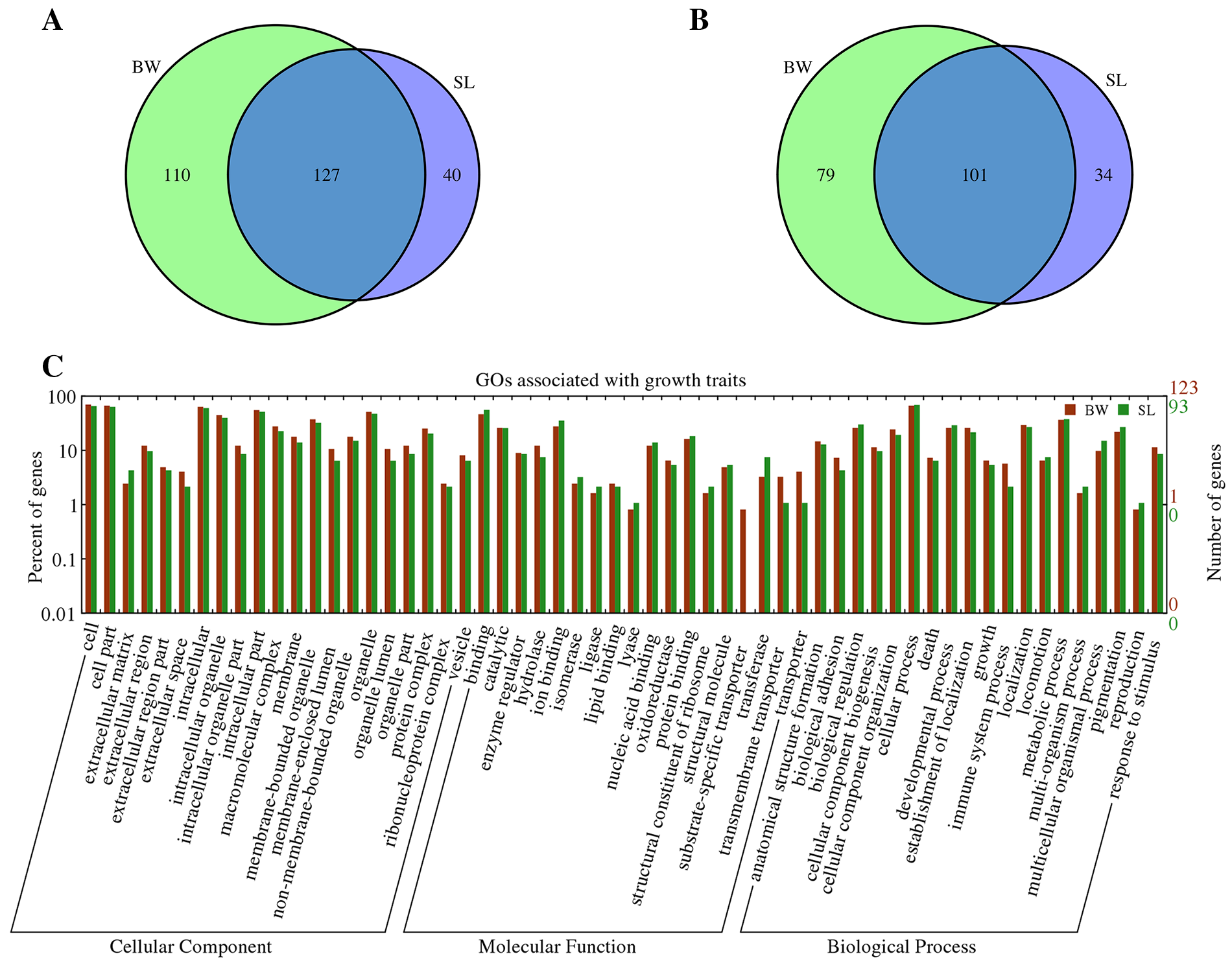


**Supplementary Figure S8. Sequences for designing primers.** Tp53-zebrafish: partial *tp53* gene sequence of zebrafish; tp53-grass_carp: partial *tp53* gene sequence of grass carp; opn1sw2-common_carp: partial *opn1sw1* gene sequence of common carp; ogg1-goldfish: partial *ogg1* gene sequence of goldfish.


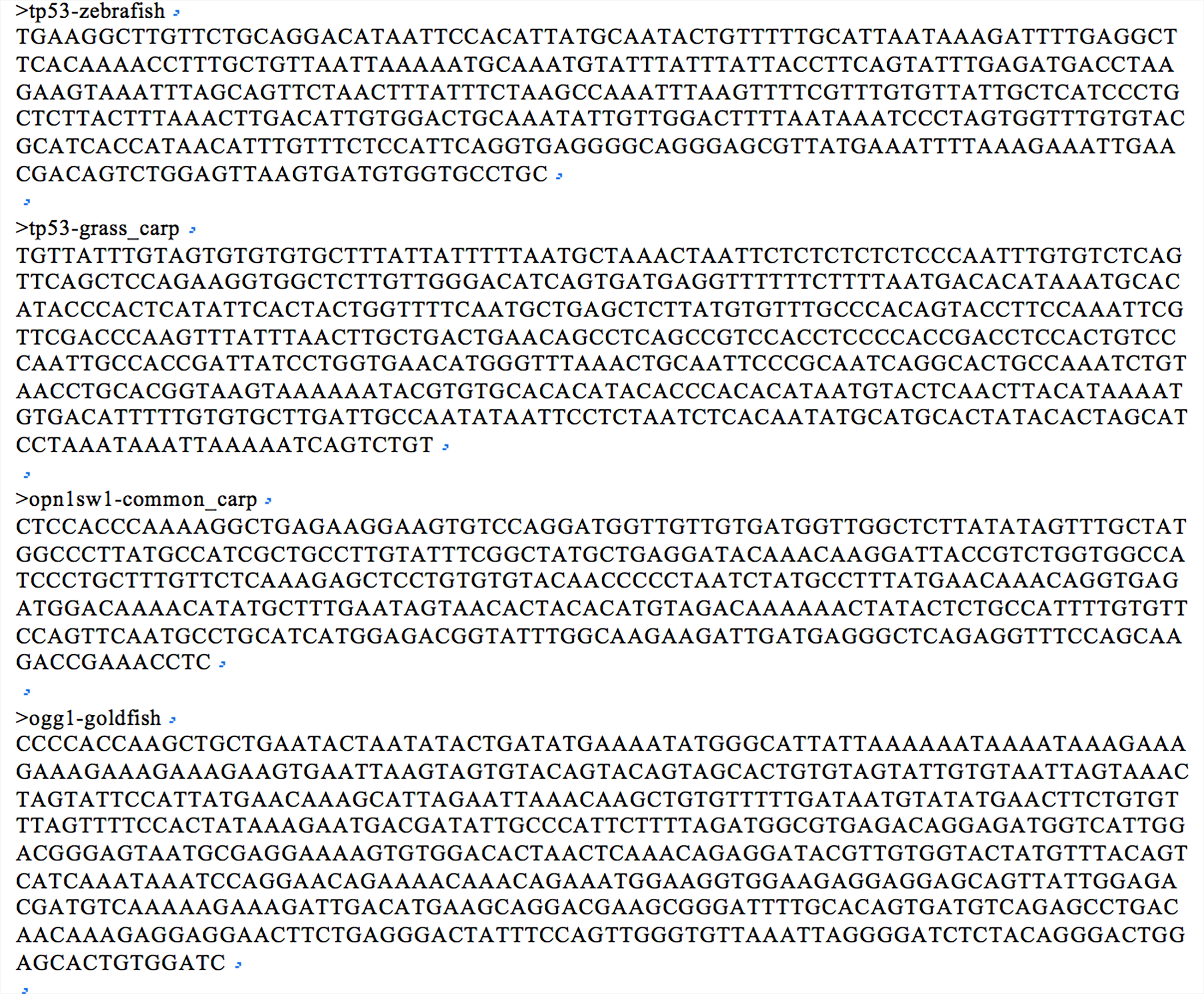


**Supplementary Table S1. Assembly of male reference transcripts**

| Transcript Number | 80,298 |
| --- | --- |
| Total bases (bp) | 61,741,462 |
| Mean transcript length | 768 |
| Maximal transcript length (bp) | 19,461 |
| Minimal transcript length(bp) | 201 |

**Supplementary Table S2. Summary for clean reads and mapping ratio of reads**

(Included in a separated excel file)

**Supplementary Table S3. Marker locations in goldfish linkage group.**

The SNP was named as transcript id followed by SNP location with separator‘_’.

(Included in a separated excel file)

**Supplementary Table S4. Blastx of goldfish marker transcripts against zebrafish proteins**

(Included in a separated excel file)

**Supplementary Table S5. Estimated haploid genome sizes using real-time PCR. Calibration curve came from *C_T_* versus log copies, m: slope, b: intercept, *R^2^*: coefficient of correlation, *CV*: coefficient of variation, *C*: *C*-value of genome.**

| Target | Sample concentration  (ng/µL) | Production length(bp) | | Calibration curve  *y=b+mx*(*R^2^*) | Target copies(copies/µL)  (mean±CV) | *C*  (pg) | Genome size(bp) |
| --- | --- | --- | --- | --- | --- | --- | --- |
|  |  | Standard | Real-time PCR |  |  |  |  |
| zebrafish(*tp53*) | 26.99 | 399 | 129 | y=32.62-3.23x(0.9987) | 17045±4.4% | 1.58 | 1.44×10^9^ |
| comm carp(*opn1sw1*) | 30.18 | 378 | 108 | y=39.24-3.17x(0.9945) | 16769±7.9% | 1.80 | 1.64×10^9^ |
| grass carp(*tp53*) | 47.86 | 334 | 164 | y=40.86-3.23x(0.9939) | 46482±7.0% | 1.03 | 9.4×10^8^ |
| goldfish(*ogg1*) | 38.82 | 313 | 108 | y=33.70-2.97x(0.9839) | 21810±3.2% | 1.78 | 1.62×10^9^ |

**Supplementary Table S6. QTL results for BW and SL**

(Included in a separated excel file)

**Supplementary Table S7. Association study results for BW and SL**

(Included in a separated excel file)

**Supplementary Table S8. GO annotations of genes associated with BW and SL**

(Included in a separated excel file)

Supplementary Table S9. Primer sequences and annealing temperature for conventional PCR and real-time PCR.

| **Target** | | **Sequence** | | **Annealing temperature** |
| --- | --- | --- | --- | --- |
| Zebrafish(*tp53*) | Conventional PCR | Forward | TGAAGGCTTGTTCTGCAGGA | 62°C |
|  |  | Reverse | GCAGGCACCACATCACTTAA |  |
|  | Real-time PCR | Forward | TGCTCATCCCTGCTCTTACT | 63.5°C |
|  |  | Reverse | CCTGCCCCTCACCTGAATG |  |
| Grass carp (*tp53*) | Conventional PCR | Forward | CAGAAGGTGGCTCTTGTTGG | 62°C |
|  |  | Reverse | ATGTGTGGGTGTATGTGTGC |  |
|  | Real-time PCR | Forward | CCAAATTCGTTCGACCCAAGT | 63.5°C |
|  |  | Reverse | GTGCAGGTTACAGATTTGGCA |  |
| Common carp  (*opn1sw1)* | Conventional PCR | Forward | CTCCACCCAAAAGGCTGAGA | 62°C |
|  |  | Reverse | GAGGTTTCGGTCTTGCTGGA |  |
|  | Real-time PCR | Forward | ATGCCATCGCTGCCTTGTAT | 65°C |
|  |  | Reverse | GGGGGTTGTACACACAGGAG |  |
| Goldfish(*ogg1*) | Conventional PCR | Forward | GGAGATGGTCATTGGACGGG | 62°C |
|  |  | Reverse | GATCCACAGTGCTCCAGTCC |  |
|  | Real-time PCR | Forward | GCACAGTGATGTCAGAGCCT | 65°C |
|  |  | Reverse | GATCCACAGTGCTCCAGTCC |  |

**References**

1. Wang, Y., Lu, Y., Zhang, Y., et al. 2015, The draft genome of the grass carp (Ctenopharyngodon idellus) provides insights into its evolution and vegetarian adaptation. *Nat. Genet.*, **47**, 625-631.

2. Howe, K., Clark, M. D., Torroja, C. F., et al. 2013, The zebrafish reference genome sequence and its relationship to the human genome. *Nature*, **496**, 498-503.

3. Xu, P., Zhang, X., Wang, X., et al. 2014, Genome sequence and genetic diversity of the common carp, Cyprinus carpio. *Nat. Genet.*, **46**, 1212-1219.

4. Untergasser, A., Cutcutache, I., Koressaar, T., et al. 2012, Primer3—new capabilities and interfaces. *Nucleic Acids Res.*, **40**, e115.

5. Wilhelm, J. 2003, Real-time PCR-based method for the estimation of genome sizes. *Nucleic Acids Res.*, **31**, 56e-56.

6. Wilhelm, J. and Hahn, M. 2004, Measuring Genome Sizes by Absolute Quantification. In: Wittwer, C., Hahn, M. and Kaul, K. (eds), *Rapid Cycle Real-Time PCR — Methods and Applications: Quantification*, Springer Berlin Heidelberg, Berlin, Heidelberg, pp. 31-41.

7. Li, H. and Durbin, R. 2009, Fast and accurate short read alignment with Burrows-Wheeler transform. *Bioinformatics*, **25**, 1754-1760.
