# Supplementary figures and images for: The genetic map of goldfish (Carassius auratus) provided insights to the divergent genome evolutions in the Cyprinidae family
Source: Sci Rep. 2016 Oct 6;6:34849. doi: 10.1038/srep34849 (PMC5052598; doi:10.1038/srep34849)

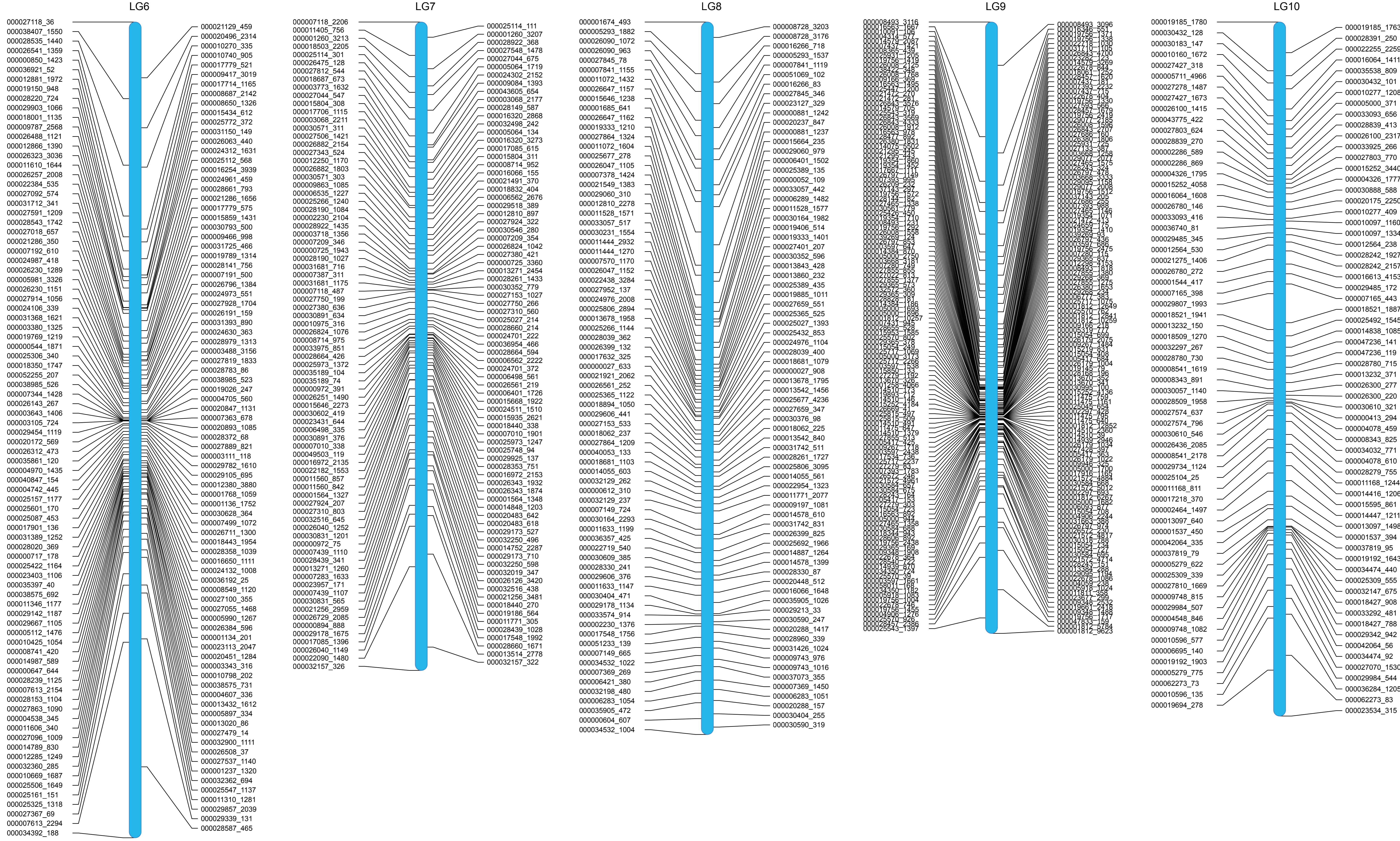

LG11

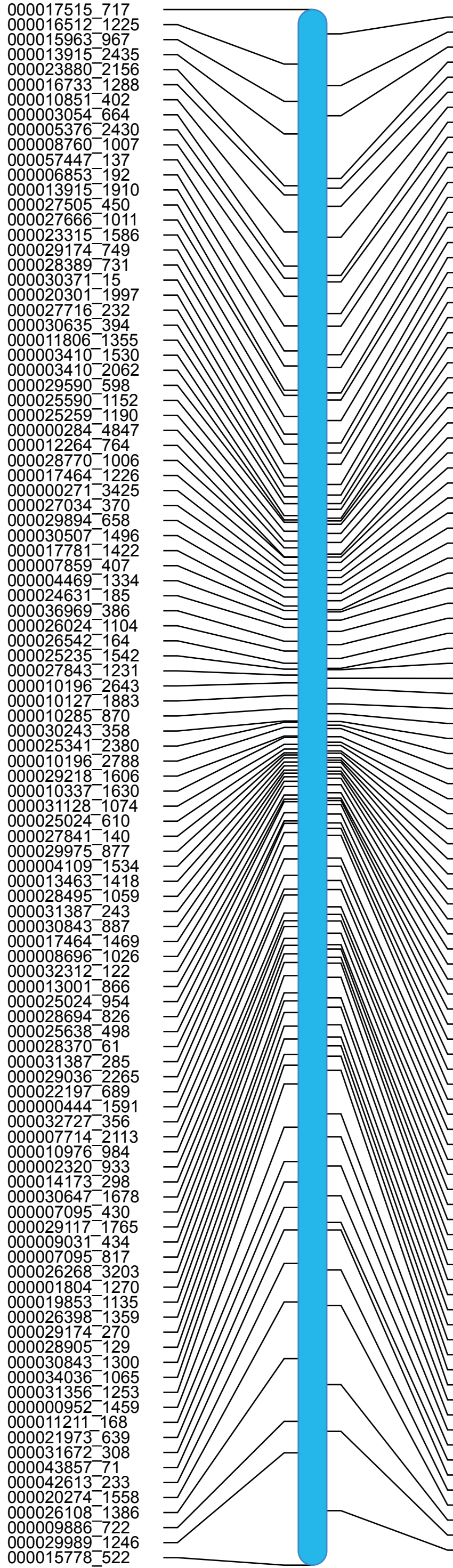

LG12

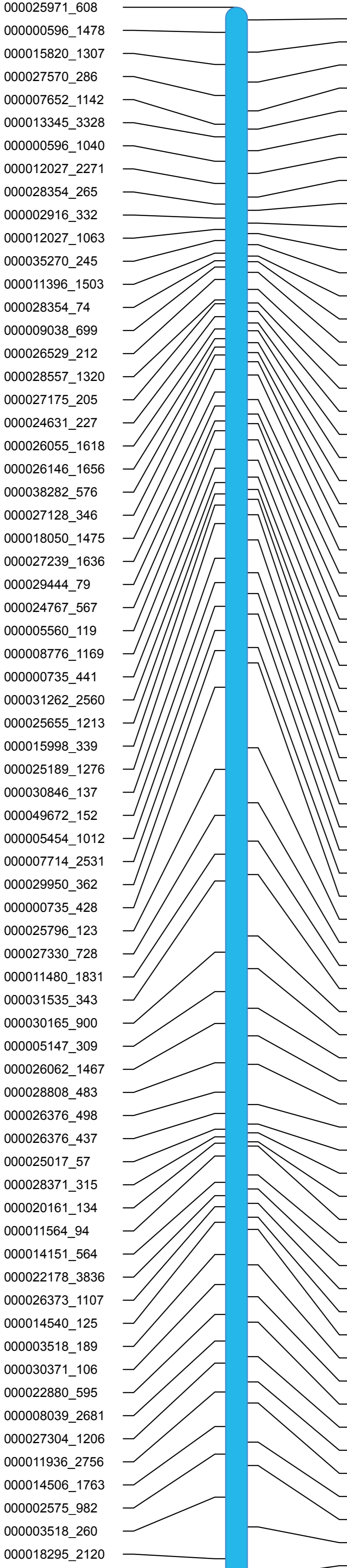

LG13

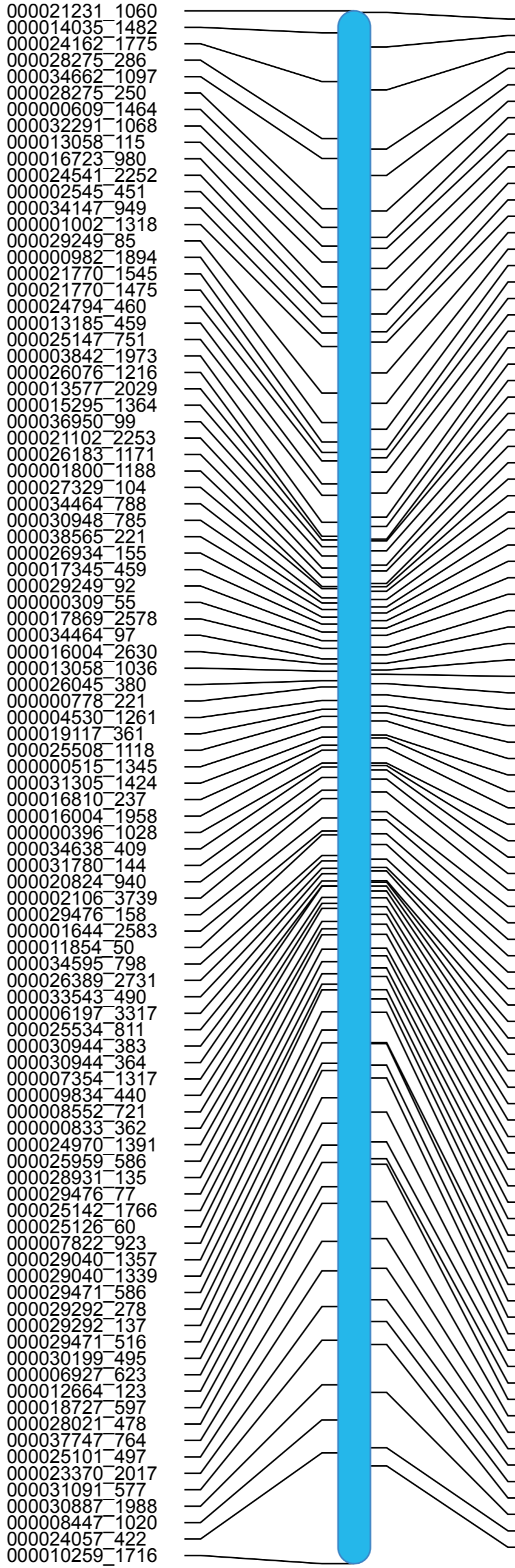

LG14

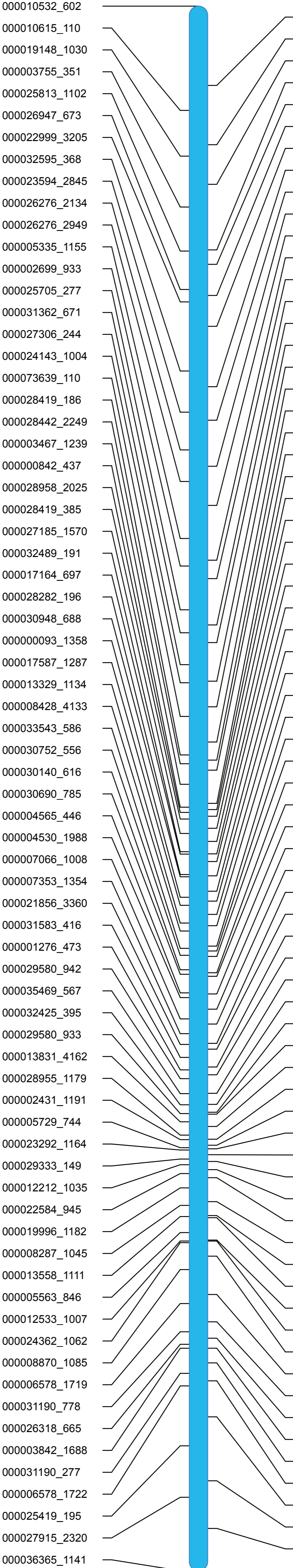

LG15

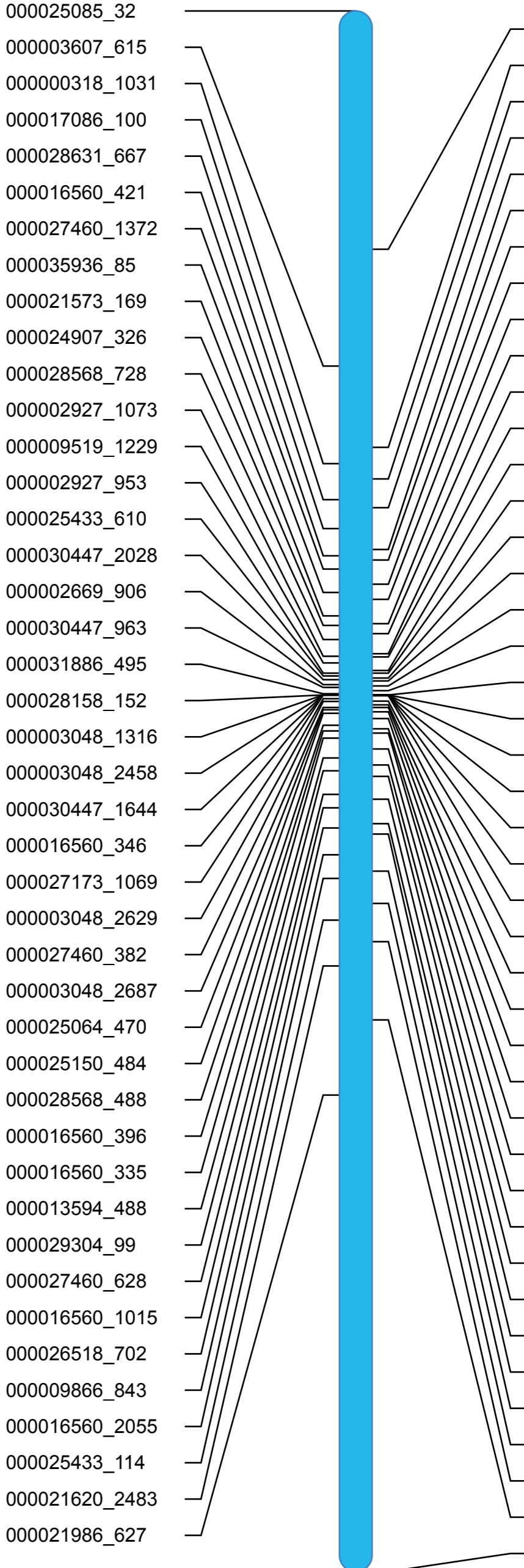



LG21

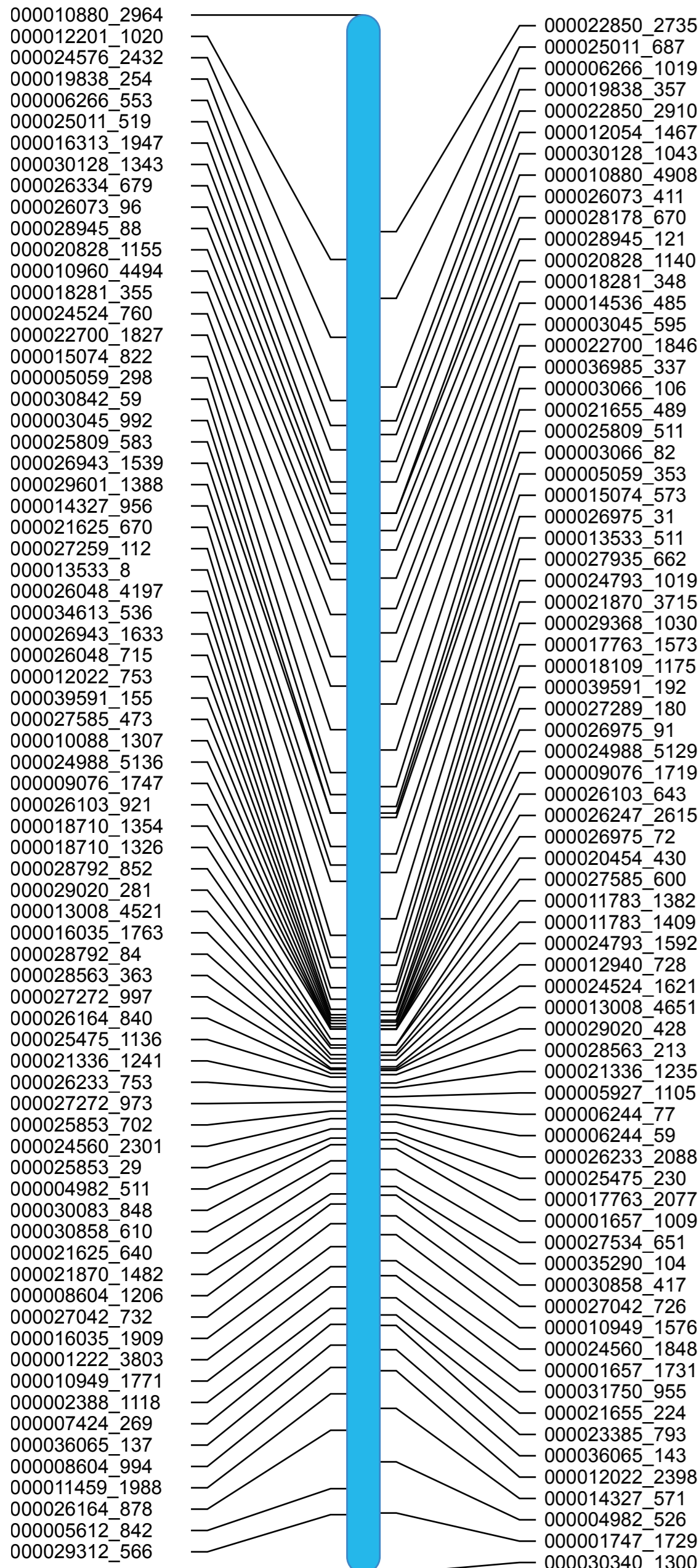

LG22

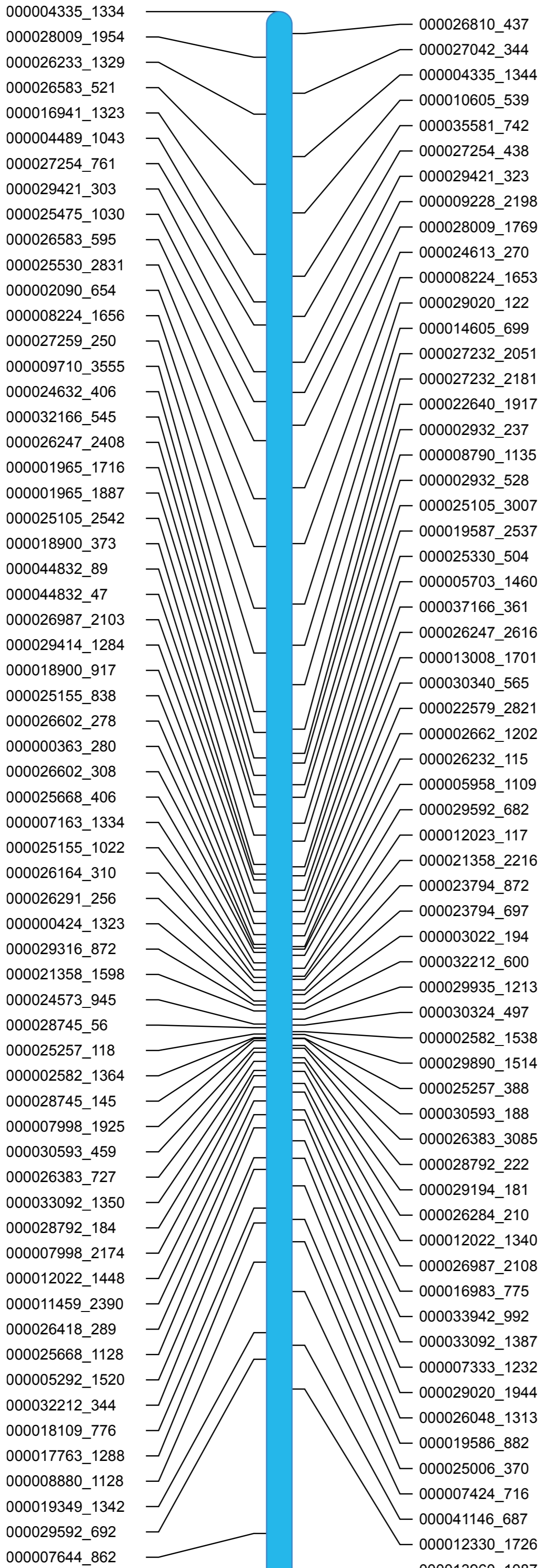

LG23

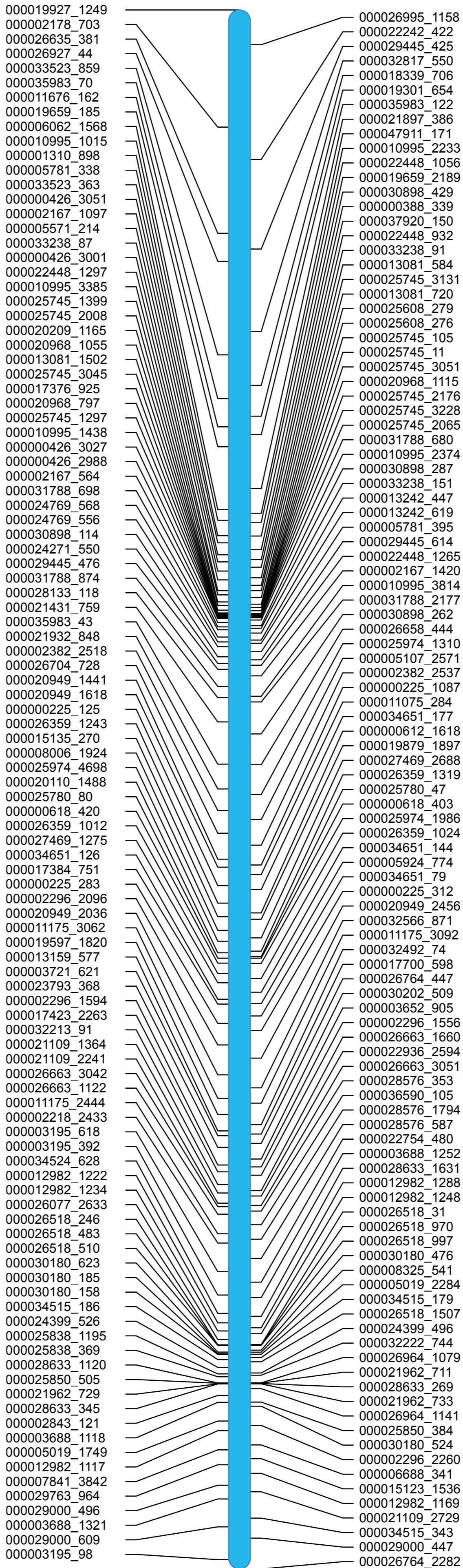

LG24

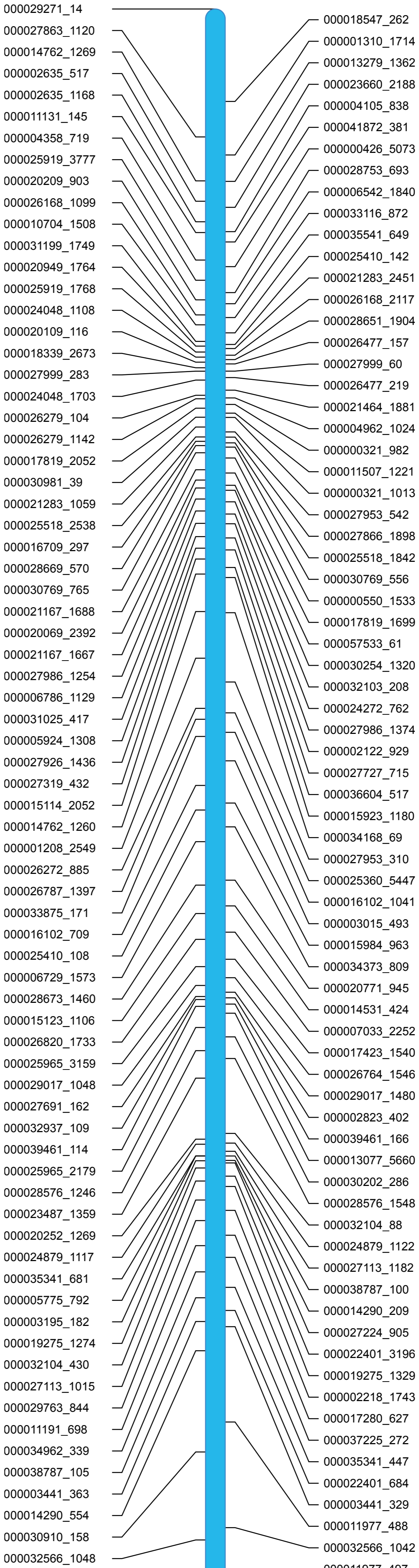

LG25

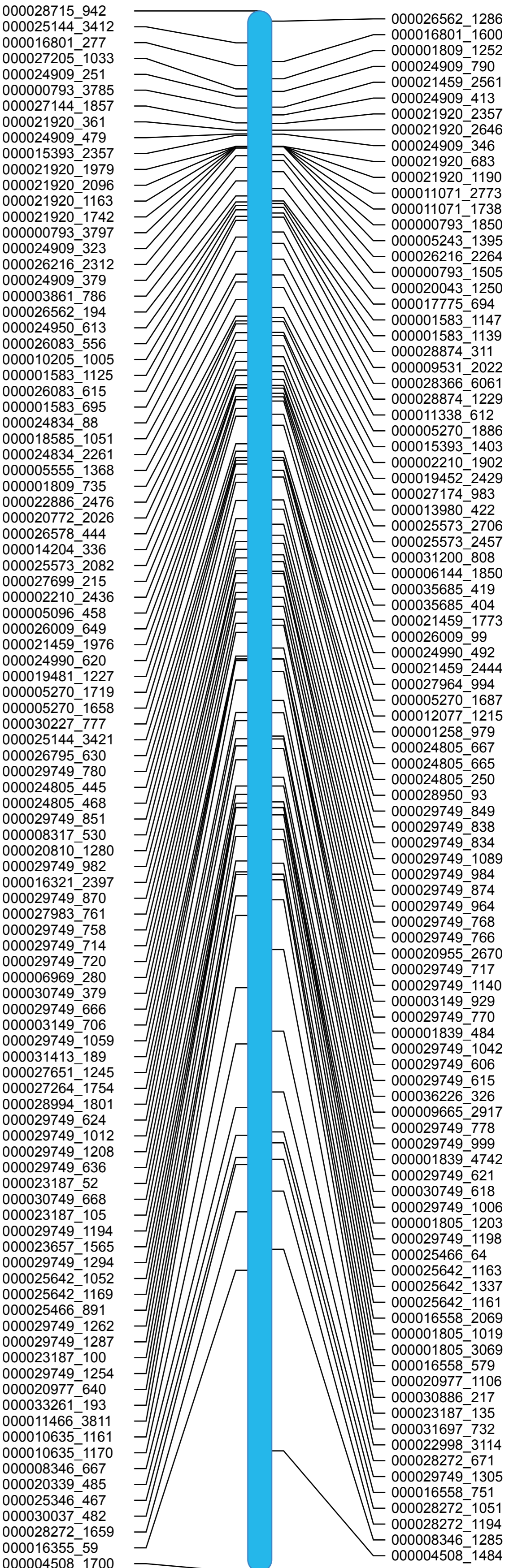





000033924, 361  
000005375, 866  
000005265, 491  
000028684, 149  
000044516, 105  
000005265, 574  
000027906, 92  
000005265, 643  
000020983, 562  
000020140, 1841  
000022517, 2253  
000012060, 5537  
000003768, 1454  
000026289, 2384  
000006977, 2834  
000009391, 2773  
000018106, 2155  
000012510, 877  
000032371, 1006  
000029140, 4604  
000014433, 2561  
000011402, 2214  
000026289, 1013  
000033301, 53  
000010975, 1905  
000001249, 2602  
000033268, 47  
000015206, 5195  
000025558, 1912  
000011067, 292  
000002403, 579  
000015206, 2074  
000015206, 3316  
000036670, 166  
000025558, 3257  
000021444, 3413  
000026591, 747  
000015206, 4790  
000021444, 2327  
000015206, 3352  
000021444, 779  
000015755, 2818  
000015206, 274  
000019846, 439  
000021444, 3526  
000018106, 2367  
000032272, 680  
000027557, 1070  
000024903, 274  
000030289, 168  
000012510, 730  
000000127, 1064  
000025558, 3132  
000026591, 420  
000025558, 3391  
000028553, 1434  
000000127, 1154  
000021235, 529  
000022482, 253  
000028138, 867  
000008282, 1551  
000000127, 1040  
000000127, 334  
000001300, 367  
000010975, 1902  
000007542, 664  
000024798, 1528  
000005908, 1700  
000036662, 156  
000026289, 1321  
000031004, 1406  
000019201, 3604  
000022079, 1601  
000023774, 1531  
0000227047, 1354  
000005910, 1834  
000023760, 720  
000002188, 671  
000009821, 1595  
000004353, 368  
000009248, 1822  
000009821, 1586  
000002188, 832  
000018226, 1445  
000012060, 5610  
000004353, 473

000018998 434  
000028412 1164  
000033924 381  
000028684 152  
00016697 490  
000029983 1477  
000027906 80  
000029083 565  
000005265 727  
00019861 681  
000022517 2265  
000030214 1359  
000001249 302  
000021494 802  
000023774 2347  
000011402 1359  
000007542 890  
000018226 770  
000029140 4600  
000144438 317  
000077032 495  
000029962 737  
000033268 76  
00011908 2280  
000021444 642  
000018106 2393  
000000127 1152  
000003819 1008  
000021444 1931  
000001249 2597  
000017466 101  
00015206 4099  
000018106 2379  
000015755 2751  
000021444 2714  
000029958 372  
000021444 2321  
000015755 2623  
000027541 1711  
000003819 1274  
000026840 171  
00019846 418  
000015755 2823  
000021444 3595  
000018106 2344  
000031004 956  
000005908 879  
000035384 415  
000000127 1115  
000035384 84  
000027557 1141  
000005908 2562  
00036670 359  
000025842 223  
000027138 534  
000026205 119  
000021235 431  
000019975 1838  
000023774 1858  
000021444 1241  
00011908 585  
000001138 267  
000000127 385  
000026293 949  
000019975 1900  
00019846 239  
000000127 1179  
000015755 3172  
000029469 116  
00018106 1928  
000019201 3081  
000014274 897  
000027099 853  
000026289 1457  
000012060 5486  
000332520 478  
000005910 1825  
000038563 293  
00018106 2968  
000002188 798  
000023507 397  
00018903 2167  
000018909 1917  
000008114 960  
000002188 818  
000013337 89

000019773, 1086  
000019469, 396  
000016546, 129  
000070040, 1274  
00001773, 1158  
000008658, 532  
000019469, 117  
000022559, 1684  
000019773, 1149  
000008989, 1963  
000011653, 777  
000019773, 1104  
000004083, 597  
000019469, 978  
000025356, 1445  
000008686, 592  
000019469, 738  
000019404, 1009  
000029919, 840  
000029919, 844  
000025852, 710  
000025852, 712  
000008986, 2276  
000019469, 1258  
000039060, 670  
0000115507, 895  
000029919, 190  
000046739, 66  
000008855, 1816  
000031360, 1639  
000031259, 795  
000025265, 105  
000028390, 1014  
000027683, 249  
000008855, 797  
000028135, 1458  
000013080, 962  
000027631, 612  
000027631, 598  
000005341, 1869  
000000142, 1369  
000022901, 1486  
000025732, 617  
000007261, 1890  
000026202, 565  
000036506, 85  
000002871, 1825  
000027305, 1209  
000039171, 244  
000006923, 4271  
000027305, 2010  
000022123, 5871  
000027168, 2352  
000034854, 241  
000028390, 815  
000008855, 1875  
000002452, 1866  
000011910, 1576  
000027785, 177  
000019047, 2202  
000027766, 634  
000030798, 956  
000027766, 660

|  |                |
|--|----------------|
|  | 000030266_671  |
|  | 000016546_1212 |
|  | 000032228_652  |
|  | 000019773_1137 |
|  | 000019469_112  |
|  | 000019773_1155 |
|  | 000019773_1167 |
|  | 000019773_1143 |
|  | 000019773_1131 |
|  | 000003450_1588 |
|  | 000019773_1146 |
|  | 000019469_536  |
|  | 000012032_1020 |
|  | 000025852_993  |
|  | 000027168_1029 |
|  | 000027269_1190 |
|  | 000007400_1898 |
|  | 000041179_459  |
|  | 000007400_1930 |
|  | 000025852_626  |
|  | 000025852_609  |
|  | 000019469_1251 |
|  | 000027930_538  |
|  | 000027097_1516 |
|  | 000003450_893  |
|  | 000036399_735  |
|  | 000007400_1973 |
|  | 000028390_619  |
|  | 000003450_915  |
|  | 000004468_1480 |
|  | 000004468_259  |
|  | 000035438_559  |
|  | 000004468_152  |
|  | 000009653_2184 |
|  | 000025476_1640 |
|  | 000012861_582  |
|  | 000025476_3857 |
|  | 000033230_359  |
|  | 000027631_622  |
|  | 000027877_603  |
|  | 000019142_320  |
|  | 000024778_526  |
|  | 000012861_951  |
|  | 000000576_769  |
|  | 000057199_167  |
|  | 000013252_409  |
|  | 000003075_2899 |
|  | 000027305_2337 |
|  | 000023952_3356 |
|  | 000030482_1781 |
|  | 000027241_101  |
|  | 000019291_931  |
|  | 000006923_4406 |
|  | 000003229_1630 |
|  | 000030344_1511 |
|  | 000027305_2345 |
|  | 000000576_1678 |
|  | 000046663_296  |
|  | 000024778_1086 |
|  | 000000142_231  |
|  | 000018015_821  |
|  | 000028390_812  |

000030765, 379  
000029783, 741  
000020050, 646  
000020719, 654  
000025688, 2612  
000026911, 315  
000027193, 657  
000033661, 426  
000033709, 148  
000020486, 1569  
000030827, 403  
000080878, 3272  
000026898, 48  
000025688, 1099  
000002261, 1147  
000028050, 737  
000027672, 75  
000027365, 222  
000027713, 389  
000018495, 774  
000027821, 73  
000030634, 1080  
000025258, 2626  
000036704, 804  
000001056, 1815  
000011721, 965  
000014874, 1350  
000023409, 1864  
000008743, 1569  
000008743, 1638  
000021885, 211  
000026010, 1224  
000036390, 128  
000028390, 1265  
000005448, 1570  
000025180, 168  
000025180, 381  
000037998, 244  
000025859, 871  
000023465, 106  
000033981, 395  
000027000, 172  
000037842, 1012  
000017455, 705  
000017260, 376  
000008855, 1660  
000010475, 173  
000010997, 399  
000010405, 502  
000006452, 721  
000017954, 65  
000035867, 336  
000017954, 570  
000012544, 377  
000010997, 280  
000014097, 104  
000002961, 3799  
000027624, 528  
000036431, 97  
000015507, 681  
000009653, 2502  
000025068, 79  
000029260, 115  
000009353, 506  
000007463, 812  
000018244, 2128  
000025187, 1325  
000007261, 1752  
000032241, 2713  
000014613, 440  
000013632, 1366

000011355\_1322

000037661\_419

000020486\_1499

000000810\_4242

000012407\_223

000031138\_755

000008078\_3116

000033079\_143

000015468\_1013

000003254\_1141

000015764\_385

000015468\_1002

000021818\_1588

000028678\_930

000021818\_1347

000013049\_201

000027365\_251

000015573\_1667

000024712\_209

000011574\_525

000030634\_1004

000002271\_1133

000009739\_1390

000046067\_103

000006756\_1825

000005448\_2161

000014874\_1038

000015042\_1617

000019887\_1031

000028913\_1001

000005341\_1765

000026010\_1185

000026189\_1108

000022565\_1332

000010537\_864

000026758\_1181

000009586\_878

000032599\_963

000009230\_1413

000033981\_106

000017455\_700

000027000\_94

000008855\_1649

000031708\_434

000024316\_613

000025859\_739

000002708\_2861

000037998\_247

000033190\_60

000006452\_539

000012544\_4013

000007281\_191

000015507\_892

000027624\_1209

000010475\_294

000032203\_1027

000027269\_1228

000035867\_546

000007526\_444

000018113\_914

000025068\_255

000011373\_1626

000036716\_667

000011638\_40

000024320\_992

000022274\_1449

000018031\_1760

000014613\_434

000029715\_1006

000019404\_1057

000035745\_215

000029111 621  
000028634 427  
000028634 427  
000026513 201  
000036169 154  
0000112241 1744  
000010182 857  
000010182 857  
000029611 1107  
000012944 619  
000000045 237  
000000045 237  
000019635 645  
000010664 673  
000009170 1164  
000014899 75  
0000181325 2933  
000032464 71  
000012152 2332  
000012152 2332  
000017341 2132  
000032985 864  
000025788 1399  
000000045 237  
000004967 581  
000028567 1217  
000004967 581  
00001866 626  
000027556 126  
000018006 1486  
000000045 237  
000012064 62  
000025468 243  
000011088 76  
000000045 237  
00003893 90  
000009424 961  
000014952 1991  
00001690 369  
000000045 237  
000052759 55  
000028302 111  
000052759 55  
000000045 237  
000013136 1035  
000031883 1035  
000021714 1511  
000000045 237  
000030084 1104  
000003870 317  
000030990 694  
000008146 323  
000008146 323  
000028577 211  
000023642 146  
000032985 963  
000000045 237  
000020831 1328

|  |            |      |
|--|------------|------|
|  | 000026087  | 335  |
|  | 000019245  | 1886 |
|  | 0000000045 | 178  |
|  | 000012944  | 830  |
|  | 00002962   | 1136 |
|  | 000010664  | 1585 |
|  | 000026798  | 1231 |
|  | 000020566  | 4200 |
|  | 000028769  | 2040 |
|  | 000029621  | 620  |
|  | 000023184  | 456  |
|  | 000030955  | 765  |
|  | 000009170  | 1176 |
|  | 000007834  | 1212 |
|  | 000030095  | 68   |
|  | 000017890  | 1246 |
|  | 000026814  | 834  |
|  | 000034012  | 249  |
|  | 000029204  | 287  |
|  | 000016126  | 503  |
|  | 000005469  | 1100 |
|  | 000029583  | 1245 |
|  | 000025110  | 1603 |
|  | 000029170  | 1117 |
|  | 000029349  | 46   |
|  | 000029170  | 1104 |
|  | 000034875  | 281  |
|  | 000030730  | 853  |
|  | 000030730  | 104  |
|  | 000009959  | 147  |
|  | 000028445  | 458  |
|  | 000000018  | 502  |
|  | 000025468  | 208  |
|  | 000021710  | 278  |
|  | 000021710  | 289  |
|  | 000033978  | 386  |
|  | 000025170  | 501  |
|  | 000021239  | 1052 |
|  | 000022788  | 543  |
|  | 0000000370 | 1319 |
|  | 0000000370 | 1181 |
|  | 000020762  | 1205 |
|  | 000028302  | 78   |
|  | 000017414  | 2832 |
|  | 000013733  | 1238 |
|  | 000020911  | 401  |
|  | 000006071  | 2578 |
|  | 000018803  | 1730 |
|  | 0000000742 | 1843 |
|  | 000029983  | 276  |
|  | 000031883  | 138  |
|  | 000018644  | 742  |
|  | 000025327  | 1951 |
|  | 000025778  | 425  |
|  | 000025778  | 1111 |
|  | 000019803  | 1354 |
|  | 000007633  | 56   |
|  | 000005605  | 1229 |
|  | 000012033  | 588  |

000026827, 491  
000015493, 1094  
000027510, 1146  
000027101, 642  
000027101, 912  
000029932, 371  
000028869, 433  
000020257, 378  
000007877, 845  
000015940, 2278  
000036626, 294  
000028634, 241  
000026625, 267  
000018679, 1345  
000021190, 1204  
000004153, 1115  
000028246, 1318  
000005247, 501  
000016477, 877  
000025417, 1197  
000062000, 554  
000028129, 1005  
000009596, 515  
000017397, 1416  
000001693, 3811  
000029120, 332  
000025327, 1930  
000030863, 494  
000001693, 3698  
000018801, 1278  
000021703, 1308  
000005391, 1611  
000009424, 810  
000017397, 1086  
000016388, 952  
000021703, 202  
000013642, 1970  
000011084, 407  
000030361, 1222  
000033502, 501  
000024204, 664  
000014456, 1000  
000027180, 2560  
000030990, 856  
000026785, 640  
000031930, 168  
000025069, 1683  
000020762, 397  
000005640, 1441  
000001672, 5419  
000019161, 3725  
00007949, 1891  
000024139, 451  
000023463, 832  
000027463, 673  
000007334, 1561  
000012106, 1400  
000008080, 1428  
000010701, 357  
000004755, 1153  
000030032, 325  
000025958, 1510  
000027451, 471  
000018513, 665  
000008786, 141  
000019832, 1246  
000024139, 163  
000002011, 293  
000031969, 1271  
000021853, 434  
000019832, 1096  
000024826, 1461  
000037216, 235  
000014871, 2419  
000029163, 161  
000007334, 2298  
000001189, 1040

000004017\_1537  
000026033\_905  
000027152\_751  
000014593\_1554  
000027510\_1139  
000027610\_554  
000028410\_1387  
000018869\_88  
000000572\_1184  
000009327\_780  
000009327\_865  
000028410\_1054  
000012912\_1508  
000028246\_1279  
000002778\_1319  
000029418\_268  
000012912\_1418  
000029932\_122  
000031443\_598  
000025848\_86  
000007834\_804  
000006200\_622  
000019656\_382  
000029204\_553  
000033435\_298  
000021190\_832  
000019011\_1390  
000019011\_1393  
000044022\_404  
000025320\_512  
000005391\_1314  
000019393\_104  
000025889\_1020  
000025437\_776  
000019393\_2209  
000000997\_847  
000016676\_2268  
000027943\_895  
000002778\_893  
000014899\_998  
000009673\_1684  
000027943\_1203  
000008186\_102  
000026420\_585  
000026785\_597  
000005763\_1618  
000020201\_1415  
000008694\_1512  
000031076\_215  
000021181\_934  
000011645\_520  
000010770\_1101  
000024611\_156  
000002267\_2864  
000023987\_450  
000002267\_1382  
000026420\_237  
000021553\_555  
000027451\_464  
000007607\_1247  
000030032\_327  
000038183\_551  
000040408\_377  
000013324\_1630  
000001672\_4809  
000008896\_1615  
000012106\_1388  
000013324\_1626  
000001648\_664  
000020065\_512  
000014871\_2414  
000004027\_2817  
000025847\_591  
000025847\_1491  
000014580\_577  
000022528\_905



LG46

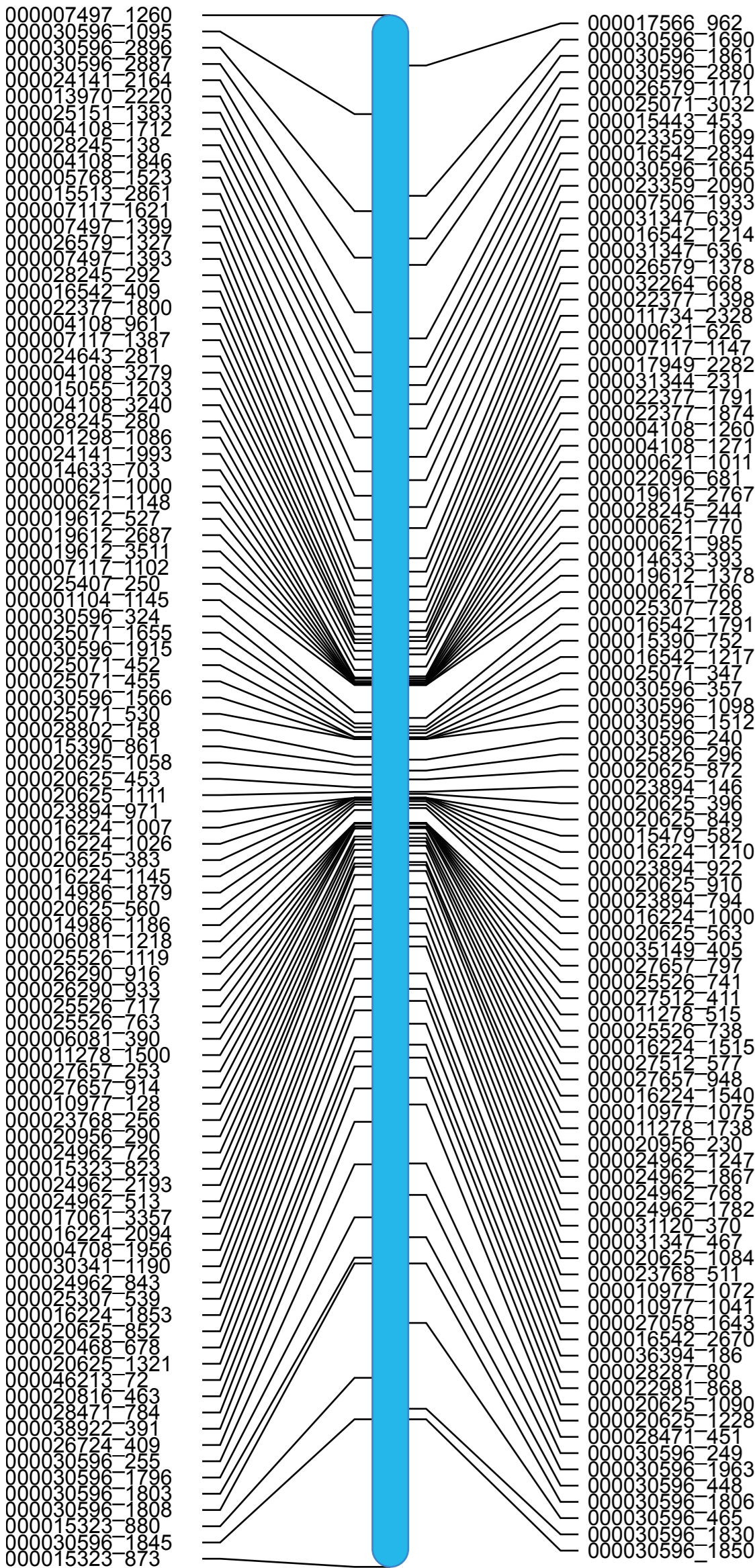

LG47

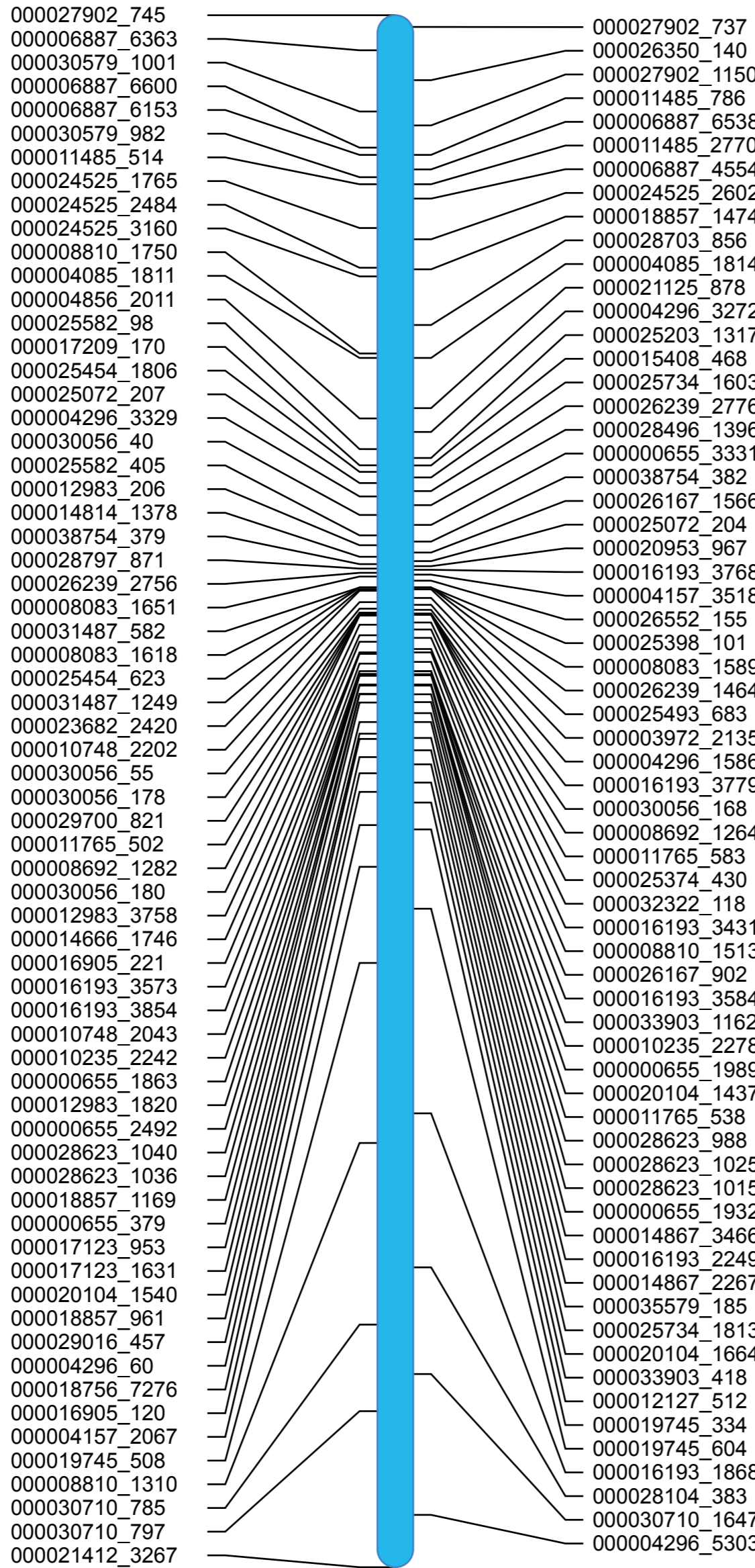

LG48

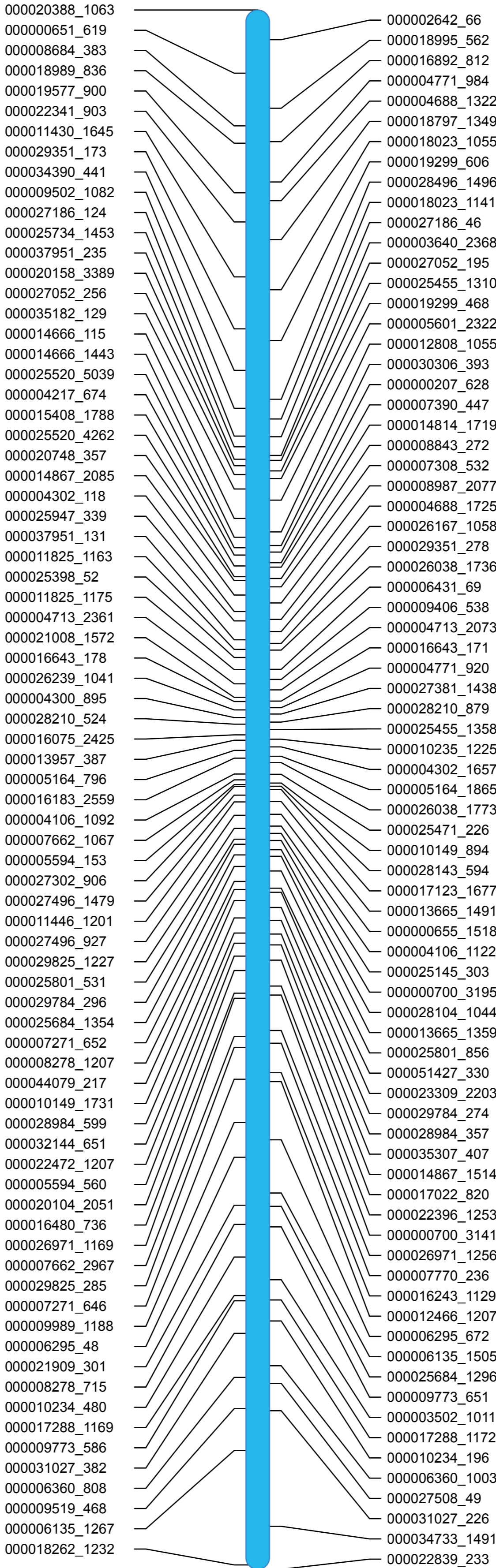

LG49

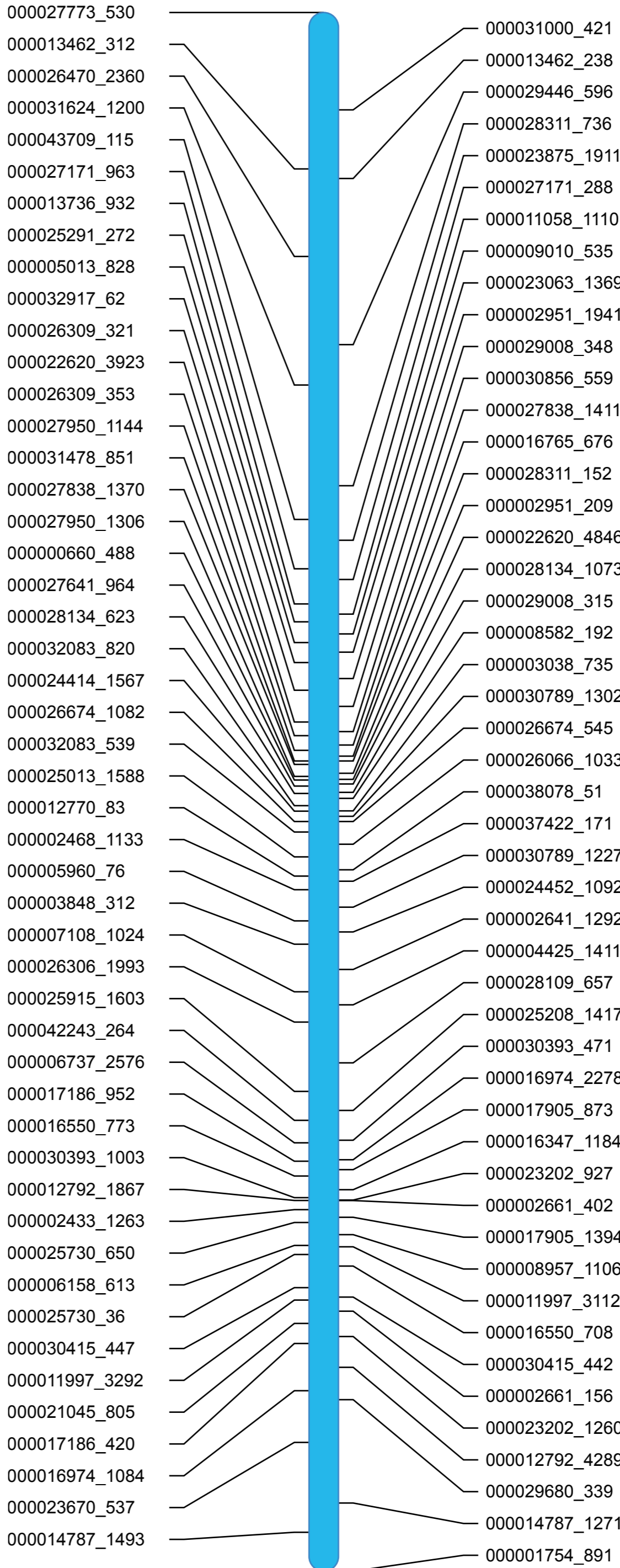

LG50

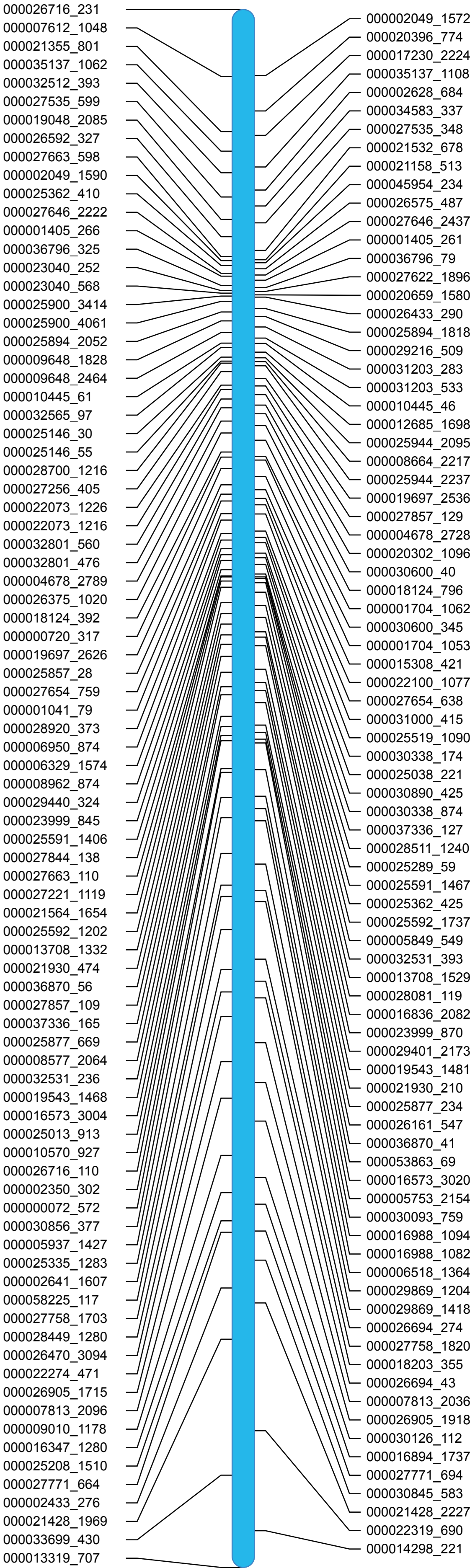

Supplement: Supplementary Figure S2 [file srep34849-s2.zip › Supplementary Figure S2.pdf]
